# *MICA* and *NKG2D* gene polymorphisms influence graft survival, and response to therapy in kidney transplantation

**DOI:** 10.3389/fimmu.2024.1440887

**Published:** 2024-11-07

**Authors:** Roberto Littera, Stefano Mocci, Davide Argiolas, Letizia Littarru, Sara Lai, Maurizio Melis, Celeste Sanna, Caterina Mereu, Michela Lorrai, Alessia Mascia, Andrea Angioi, Giacomo Mascia, Valeria Matta, Nicola Lepori, Matteo Floris, Cristina Manieli, Paola Bianco, Daniela Onnis, Stefania Rassu, Silvia Deidda, Mauro Giovanni Carta, Erika Giuressi, Andrea Perra, Luchino Chessa, Sabrina Giglio, Antonello Pani

**Affiliations:** ^1^ Medical Genetics, R. Binaghi Hospital, Cagliari, Italy; ^2^ AART-ODV (Association for the Advancement of Research on Transplantation), Cagliari, Italy; ^3^ Medical Genetics, Department of Medical Sciences and Public Health, University of Cagliari, Cagliari, Italy; ^4^ Oncology and Molecular Pathology Unit, Department of Biomedical Sciences, University of Cagliari, Cagliari, Italy; ^5^ Center for Research University Services (CeSAR), (Centro Servizi di Ateneo per la Ricerca), University of Cagliari, Monserrato, Italy; ^6^ Nephrology, Dialysis and Transplantation Unit, ARNAS “G. Brotzu”, Cagliari, Italy; ^7^ Department of Medical Science and Public Health, University of Cagliari, Nephrology, Dialysis and Transplantation Unit, ARNAS “G. Brotzu”, Cagliari, Italy; ^8^ U.O. Anatomia Patologica, P.O. San Michele, ARNAS “G Brotzu”, Cagliari, Italy; ^9^ Pneumology Unit, R. Binaghi Hospital, ASSL Cagliari, Cagliari, Italy; ^10^ Department of Medical Sciences and Public Health, University of Cagliari, Cagliari, Italy; ^11^ Liver Unit, Department of Internal Medicine, University Hospital of Cagliari, Cagliari, Italy; ^12^ Department of Medical Science and Public Health, University of Cagliari, Nephrology, Dialysis and Transplantation Unit, ARNAS “G. Brotzu” Cagliari, Consiglio Nazionale delle Ricerche, Cagliari, Italy

**Keywords:** kidney transplant, antibody-mediated rejection, *MICA*, *NKG2D*, DSA

## Abstract

**Background:**

Antibody-mediated rejection is a significant cause of kidney transplant failure. Recent studies have shown that the MHC class I *MICA* gene influences the transplantation outcome. However, the role of the primary *MICA* receptor, NKG2D, has yet to be explored.

**Aim:**

We aimed to investigate the correlation between recipient/donor *MICA* allele matching and *NKG2D* genotype with the risk of antibody-mediated rejection and their potential clinical effects and implications for organ maintenance therapy.

**Methods:**

Of the 524 patients who underwent transplantation, 387 were eligible for the study. Complete *MICA* allele and two functional polymorphisms of *NKG2D* (*rs1049174C>G* and *rs2255336G>A*) were analyzed in 148 transplanted patients and 146 controls.

**Results:**

Increased recipient/donor *MICA* allele mismatches correlate with an elevated risk of antibody-mediated rejection (X^2^ = 6.95; Log-rank=0.031). Notably, the *rs1049174[GG]* genotype contributes to a significantly increased risk of antibody-mediated rejection (X^2^ = 13.44; Log-rank=0.001 and *X* ^2^ = 0.34; Log-rank=0.84). The combined effect of two *MICA* allele mismatches and *rs1049174[GG]* genotype shows the highest risk (X^2^ = 23.21; Log-rank<0.001). Most importantly, patients with *rs1049174[GG]* and *rs2255336[*AA*]* genotypes may respond less to mTOR inhibitor immunosuppressive therapy than Calcineurin inhibitors (*rs1049174[GG];* P=0.035; and *rs2255336[AA]*; P=0.002).

**Conclusion:**

Recipient/donor *MICA* allele mismatches and specific *NKG2D* variants, as well as their combinations, influence kidney transplant outcomes, providing insights for personalized treatment and enhancing graft survival.

## 1 Background

Kidney transplantation is the best treatment for kidney function in patients with advanced chronic kidney disease or end-stage kidney disease (ESKD) (1, 2). Transplantation significantly reduces overall mortality and improves the quality of life for patients with renal disease (3, 4). However, kidney transplantation is a complex procedure that relies on several pivotal immunological and non-immunological factors that directly affect the graft’s survival and functionality (5, 6).

Antibody-mediated rejection is undoubtedly the most important factor that adversely affects the survival of the transplanted kidney in the medium and long term (7, 8).

Antibody-mediated rejection (ABMR) constitutes organ injury triggered by circulating donor-specific antibodies (DSA), which can target either human leukocyte antigens (HLAs) or non-HLA antigens (9). Identifying ABMR typically involves assessing the levels of DSAs and performing a kidney biopsy that reveals features such as microvascular inflammation (MVI) and C4d deposition in the endothelium (10–12).

While the compatibility of HLA molecules between donor and recipient has historically been the main focus in kidney transplantation, recent studies suggest that incompatibilities at other loci, such as *MICA* (major histocompatibility complex class I-related chain A) and *KIR* genes and *NKG2D* (natural killer group 2 member D) (*KLRK*), can influence graft outcome (13–17).

MICA acts as a ligand for NKG2D, an activating receptor expressed on NK cells, NKT cells, γδ T cells, and CD8+ αβ T cells (1, 18, 19). The binding of MICA ligands to NKG2D activates NK cells, enhances their functions, and allows them to function as a bridge between innate and adaptive immunity (20).

The pathogenic role of MICA-specific antibodies in kidney transplantation remains controversial. However, recent studies indicate that patients with *MICA* mismatches exhibit notably diminished graft survival compared to those with *MICA*-matched donors, with respective five-year graft survival rates of 88% and 96% (15). Additionally, genetic variability of *NKG2D* also may influence the receptor’s functional capacity, with at least two haploblocks identified to affect receptor expression activity levels (21).

This study aims to 1) evaluate the impact of *MICA* polymorphisms on kidney transplantation, focusing on the incidence of antibody-mediated rejection and graft function survival in the Sardinian population 2) Investigate how *NKG2D* polymorphisms in combination with *MICA* compatibility may influence immunological activity and assess their immunological significance relative to the classic *HLA* system.

## 2 Materials and methods

### 2.1 Study population

**Figure 1:**
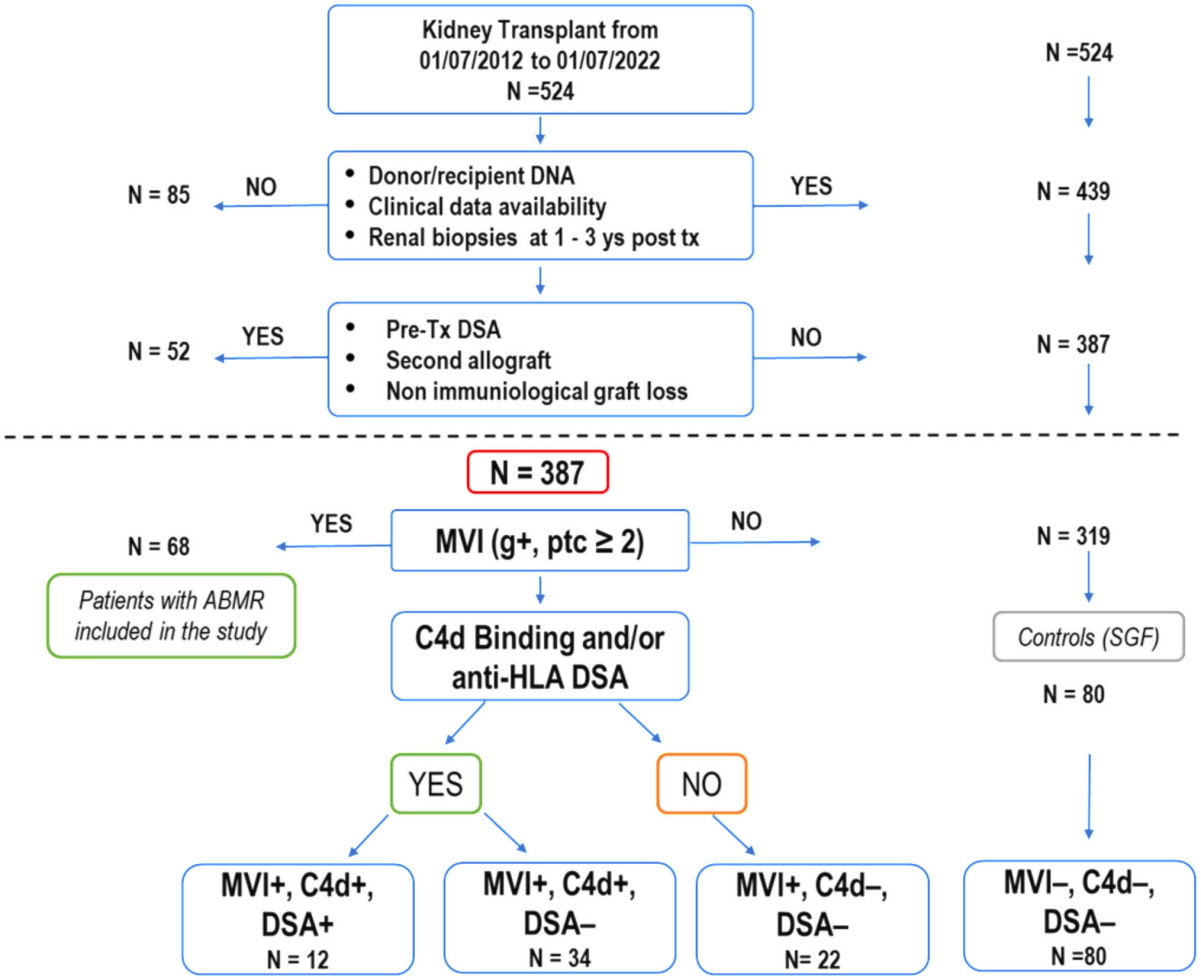
Selection process and characteristics of kidney transplant recipients in the study. Out of 524 patients who underwent kidney transplants over ten years, 387 were enrolled for analysis based on specific criteria, including renal biopsies at 1 and 3 years after transplantation (as required for post-transplant follow-up by the Cagliari transplant center), absence of second transplant and pre-transplant donor-specific HLA antibodies. Among these, 68 patients (21.3%) experienced a progressive decline in graft function attributed to antibody-mediated rejection (ABMR), with histological confirmation through renal biopsies. Some of these patients showed a mixed histological picture of ABMR and TCMR. The 68 patients with ABMR presented with a histological picture of MVI+ (g+, ptc ≥ 2). They were divided into three subgroups based on the presence or absence of C4d and the presence or absence of DSA (MVI+, C4d+, DSA+;MVI+, C4d+, DSA-; MVI+, C4d-, DSA-). The remaining 319 patients never exhibited clinical, histological, or laboratory signs of organ damage. Eighty patients (MVI-, C4d-, DSA-), randomly selected, were used as a control group (SGF). (ABMR, antibody-mediated rejection; DSA, donor-specific HLA antibodies; MVI, microvascular inflammation; TCMR, T-cell-mediated rejection).

From July 2012 to July 2022, 524 patients underwent kidney transplantation at the Organ Transplantation Center of the G. Brotzu Hospital in Cagliari, Italy. We excluded 1) cases lacking patient/donor biological material or incomplete clinical data. 2) patients who underwent a second transplant or had pre-transplant donor-specific *HLA* antibodies (pre-Tx DSA) (**Figure 1**).

Of the remaining 387 eligible patients, 68 manifested ABMR while 319 showed normal renal function. Out of these 319, 80 patients were randomly selected to be used as the stable graft function (SGF) control group.

Moreover, the total number of SGF and ABMR patients (148) was compared with a healthy cohort of 146 individuals of Sardinian descent and underwent a systematic evaluation of clinical and immunological parameters significantly impacting transplantation outcomes. These parameters included *HLA* class I (0-2 *HLA-A*, 0-2 *HLA-B*, 0-2 *HLA-C*) mismatches (score 0-6), *HLA* class II (0-4 *HLA-DRB1, HLA-DQB1*) mismatches (score 0-4), recipient *NKG2D* (*KLRK1*) polymorphisms *rs1049174* and *rs2255336*, *MICA* mismatches (score 0-2). The analysis of *MICA* alleles matching was performed considering two categories of mismatches: i) mismatches in the host-versus-graft (HvG) direction where the donor but not the recipient is mismatched, and ii) all types of mismatches, independent of their directions. Additional factors examined included panel-reactive antibodies (PRA) exceeding 5-10%, the duration of graft cold ischemia, the type of induction therapy received, and the immunotherapy administered post-transplant.

### 2.2 Allograft pathology

Kidney biopsies were performed when clinically justified due to suspected graft dysfunction, such as unexplained changes in graft function, new-onset proteinuria, rising serum creatinine, or other deviations from the standard post-transplantation course. These biopsies were carried out to promptly detect and address any potential pathological alterations affecting graft function. Each specimen was assessed according to the standards from the 15th Banff Conference held in 2019 (22). All samples were reviewed by three independent renal pathologists. The analysis included H&E, PAS, AFOG, and silver stain, along with immunofluorescence for IgG, IgA, IgM, kappa, lambda, C3, C4, C1q, and albumin, and immunohistochemistry for C4d, with additional stains as required (e.g., SV40).

### 2.3 Donor-specific antibody

The presence of anti-MICA and anti-HLA class I and II antibodies were determined in all pre-transplant patients. The presence of anti-HLA DSA precluded kidney transplantation at our center. Anti-HLA and MICA DSA levels: This assessment was conducted using LAB Screen Single Antigen kits from One Lambda, following the manufacturer’s instructions. Beads with a normalized MFI above 2500 for HLA or 1000 for MICA were considered positive as reported in other work (23). According to the transplant center’s operational standards, antibody testing was performed during the one-year post-transplant follow-up, or earlier if the patient showed clinical signs of rejection (renal injury/graft impairment) within the first year.

### 2.4 Genetic analysis

*HLA and MICA typing.* Genomic DNA (gDNA) was isolated from peripheral blood using QIAcube (Qiagen, Hilden, NW, Germany) and the DNA Blood Mini kits according to the manufacturer’s instructions. Isolated gDNA was HLA genotyped using the AlloSeq Tx17 assay (Care Dx, Brisbane, CA) following the recommended protocol. The AlloSeq Tx17 assay incorporates 17 specific probes designed to target *HLA-A, HLA-B, HLA-C, HLA-E, HLA-F, HLA-G, HLA-H, HLA-DRB1, HLA-DRB3, HLA-DRB4, HLA-DRB5, HLA-DQA1, HLA-DQB1, HLA-DPA1, HLA-DPB1, MICA, and MICB loci*. Library quantification was performed using the Qubit™ dsDNA HS Assay (Life Technologies, Carlsbad, California, USA), and sequencing was performed on the Illumina MiSeq platform (San Diego, CA) with 2x150 sequencing. *MICA* allele matching/mismatching was analyzed based on the recipient-versus-graft direction, where the donor, but not the recipient, is mismatched. The codon position 129 of the *MICA* gene was considered to determine the presence of Methionine (Met) or Valine (Val) (*rs1051792*) and to evaluate recipient/donor *MICA* mismatches. Allele frequencies of *MICA* were calculated using direct gene counting.

#### 2.4.1 *NKG2D rs1049174* and *rs2255336* sequencing

Primer sets targeting specific regions were designed with the assistance of Primer3 version 4.1.0 (24). The annealing temperature was optimized for each primer set. The two SNPs located within the *NKG2D* (*KLRK1*) gene *rs1049174* and *rs2255336* belong to two different haplotype blocks (*NKG2D* hb-1 and hb-2), each of which generates two major haplotypes associated with low (LNK) and high natural cytotoxic activity (HNK) phenotypes as shown in **Table 1** (21). Several studies have demonstrated that high and low natural cytotoxic activity haplotype alleles (HNK1 or LNK1) belonging to *NKG2D* haplotype blocks 1 (hb-1) may be successfully predicted by only a single SNP (dbSNP: *rs1049174*) and haplotype blocks 2 (hb-2) by dbSNP *rs2255336* (25, 26). Primers are reported in the **Supplementary Table S1**. The PCR was performed according to the protocol supplied with AmpliTaq Gold™ DNA Polymerase (Applied Biosystems/Thermo Fisher Scientific, Waltham, MA). Sequencing was performed using the BigDye™ Terminator v3.1 Cycle Sequencing Kit (Applied Biosystems, USA), with the same primers described previously and cleaned up with CleanSEQ Dye-Terminator Removal Kit (Beckman Coulter, Inc.). Capillary electrophoresis was performed on the ABI 3500 Genetic Analyser (Applied Biosystems), and sequences were analyzed with Sequencher 5.3 (^©^ 2017 Gene Codes Corporation).

**Table 1:** Haplotype blocks (NKG2D hb-1 and hb-2) are split into low and high natural cytotoxic activity haplotypes.

Haplotype	dbSNP	Allele (Low)	Allele (High)
*NKG2D* hb-1	*rs1049174* G>C	C	G
*NKG2D* hb-2	*rs2255336* A>G	G	A

### 2.5 Statistical analysis

Summary statistics were calculated for the clinical and biochemical data of patients diagnosed with or without antibody-mediated rejection: interquartile ranges (IQR), medians, means, standard deviations (SD), and mean differences were calculated on all continuous variables; percentages and odds ratios (OR) were calculated on categorical data. P values and 95% confidence intervals (95% CI) were obtained using Student’s t-test or Fisher’s exact test, as appropriate. Statistical analysis was performed by using R software version 4.3.2 (27). The frequencies of *rs1049174 G>C* and *rs2255336 A>G* SNPs in the *NKG2D* gene and *MICA* alleles were compared between patients with ABMR, stable graft function (SGF), and an appropriate group of Sardinian healthy controls. The Hardy-Weinberg Equilibrium (HWE) of the SNPs and allele frequencies was examined by computing X^2HWE^ and P values. Deviation from HWE was assessed using HaploView 4.2 software (28). The linkage disequilibrium (LD) between the *rs1049174 G/C* and *rs2255336 A/G* haplotypes of the *NKG2D* gene was evaluated in transplanted patients and healthy group controls. The observed and expected frequencies in each sample were compared using the chi-square test. LD was measured by the parameters D (difference between the observed and expected frequencies) and D′ (i.e., D normalized to one: -1 ≤ D′ ≤ 1). D′ was obtained using the normalization formulas proposed by Lewontin (29) for two-loci haplotypes. We also computed the parameter *r^2^* expressing the correlation between the alleles at two loci. To compare the LD in the control and patient groups or the SGF and ABMR cohorts, we evaluated the P value associated with the chi-square variable (with two degrees of freedom) given by the difference between the chi-square variables in the two groups (with one degree of freedom). Kaplan-Meier curves were used to illustrate the cumulative incidence of antibody-mediated rejection from the date of transplantation to the date of clinical, histopathological, and immunohistochemical detection or the date of the last follow-up or death with a functioning graft. Transplant recipients were stratified into several groups according to genotypes and allele mismatches. The log-rank test was used for comparisons of the different gene profile combinations. Serum creatinine levels and glomerular filtration rate were measured at 1, 6, 12, 36, and 72 months from the date of transplantation. Comparison between groups of stratified patients was performed by computing the area under the curve (AUC) for the corresponding plots (30). AUC was evaluated using the trapezium formula extended to include all times at which serum creatinine and glomerular filtration rate (eGFR) were measured. The Student’s t-test was used to confirm statistical significance. A multivariate analysis was conducted to determine the independence from donor age and gender of the other clinical and genetic variables influencing rejection incidence and graft function. In the multivariate comparison between patients with SGF and ABMR, a logistic regression model was used to compute P values (P_M_), odds ratios (OR_M_), and 95% confidence intervals (95% CI_M_) adjusted accordingly to age and gender and for the potential confounder. The analysis included cold ischemia time, R/D *MICA* alleles full mismatch (*MICA 2MM*), allelic mismatches R/D *HLA*-*DRB1, HLA-DQB1* (*HLA II Class 1-2MM*) and HLA class I (HLA-A, -B, -C > 3*MM*), genotypes *NKG2D rs2255336 AA* and *NKG2D rs1049174 GG*, *de novo* DSA HLA Class I and II, and the combination of *MICA 2MM* with the genotype *NKG2D rs1049174 GG*, which was confirmed to be strongly associated with rejection incidence. The *de novo* DSA HLA Class I observations in the two groups of patients were too few to yield fully reliable results for OR_M_ and 95% CI_M_.

## 3 Results

### 3.1 Patient selection algorithm

Between July 2012 and July 2022, 524 kidney transplant patients (KTPs) were treated at the Brotzu transplant center in Cagliari (**Figure 1**). Eighty-five patients (16.2%) were excluded due to insufficient patient/donor biological material or incomplete clinical data, including the lack of renal biopsy in the 1st and 3rd year of post-transplant follow-up. To accurately evaluate the immunological impact of allelic *MICA* mismatch and *NKG2D* genotype on long-term graft function, we further excluded 52 patients (9.9%) who underwent a second transplant or had pre-transplant donor-specific HLA antibodies (pre-tx DSA). Out of 387 patients enrolled in the study, 68 (17.6%) had clinically manifested a progressive decline in graft function attributed to antibody-mediated rejection (ABMR), characterized by MVI+ (Banff score: g+, ptc ≥ 2) as evidenced in all cases through renal biopsies. They were divided into three subgroups based on the presence or absence of C4d+ and the presence or absence of DSA (12 patients were MVI+, C4d+, DSA+; 34 were MVI+, C4d+, DSA-; and 22 were MVI+, C4d-, DSA-). The presence of calcineurin inhibitor toxicity, hypertensive damage, BK virus, and bacterial infections was ruled out. All patients were compliant with post-transplantation immunosuppressive therapy. Three hundred nineteen patients, who showed no clinical, histological, or laboratory signs of organ damage, were included in the control group (SGF), which consisted of 80 randomly selected patients (MVI-, C4d-, DSA-).

### 3.2 Clinical characteristics of transplanted patients and donors

The age, sex, clinical, and demographic characteristics of recipient-donor pairs are detailed in **Table 2**. No significant differences were observed between the two groups of patients with SGF or ABMR in terms of the age and gender of the recipients. The number of *HLA* Class I (*HLA-A, -B, -C*) mismatches (0-6), HLA Class II (*HLA-DRB1, HLA-DQB1*) mismatches (0-4), and the percentage of sensitized patients (PRA > 5%) (31), showed no substantial differences between the SGF and ABMR groups. Significantly, the cold ischemia time in the ABMR patient group was notably more protracted than in the SGF group (747.5 **±** 211.5 vs. 590.3 **±** 22.9, OR = 157.2, 95% CI 74.0 – 590.3; P = 3.0 x 10^-4^).

**Table 2:** Clinical and immunological characteristics of transplant patients.

	Total (N=148)	SGF (N=80)	ABMR (N=68)	P-value	OR or x2-x1^a^ (95% CI)
Clinical characteristics at time of transplantation					
Recipient					
Age, years, median (IQR)	55.7 (47.0 – 65.0)	54.7 (47.5 – 63.0)	56.8 (46.5 – 69.0)	0.368	2.1 (-2.5; 6.7)
Male, n (%)	65 (43.9)	31 (38.8)	34 (50.0)	0.187	1.6 (0.8 – 3.2)
Causes for renal insufficiency, n (%)					
Hypertension or renal vascular disease, n (%)	55 (37.2)	31 (38.8)	24 (35.3)	0.734	0.9 (0.4 – 1.8)
Glomerulonephritis, n (%)	42 (28.4)	21 (26.2)	21 (30.9)	0.585	1.3 (0.6 – 2.7)
Other, n (%)	51 (34.4)	28 (35.0)	23 (33.8)	1	0.9 (0.5 – 1.9)
Donor					
Donor age, years, median (IQR)	45.0 (31.0 – 58.0)	37.5 (26.8 – 47.0)	51.0 (42.0 – 62.0)	**1.2·10^-6^**	12.7 (7.8 – 17.6)
Male donors, n (%)	98 (65.5)	62 (76.3)	36 (52.9)	**0.003**	0.3 (0.2 – 0.7)
Deceased, n (%)	144 (97.3)	79 (98.8)	65 (95.6)	0.334	0.3 (0.0 – 3.5)
Cytomegalovirus serostatus (r/d), n (%)					
negative/negative	4 (2.7)	2 (2.5)	2 (2.9)	1	1.2 (0.1 – 16.7)
negative/positive	16 (10.8)	10 (12.5)	6 (8.8)	0.598	0.7 (0.2 – 2.2)
positive/negative	24 (16.2)	16 (20.0)	8 (11.8)	0.189	0.5 (0.2 – 1.4)
positive/positive	104 (70.3)	52 (65.0)	52 (76.5)	0.151	1.7 (0.8 – 3.9)
Immunologic characteristics at time of transplantation					
HLA compatibility, (mean ± SD)					
Class I (HLA-A, B, C) allelic mismatch (0-6)	4.04 **±** 1.28	4.24 **±** 1.28	3.88 **±** 1.26	0.088	-0.36 (-0.77; 0.05)
Class II (*HLA-DRB1, -DQB1*) allelic mismatch (0-4)	1.07 **±** 0.72	1.10 **±** 0.67	1.03 **±** 0.80	0.563	-0.07 (-0.31; 0.17)
PRA (> 5%), n (%)					
Anti-HLA Class I	16 (10.8)	6 (7.5)	10 (14.7)	0.190	2.1 (0.7 – 7.5)
Anti-HLA Class II	19 (12.8)	7 (8.8)	12 (17.6)	0.140	2.2 (0.7 – 7.1)
Anti-HLA Class I and II	4 (2.7)	1 (1.3)	3 (4.4)	0.334	3.6 (0.3 – 193.6)
Cold ischemia time, minutes (mean ± SD)	660.2 **±** 230.6	590.3 **±** 222.9	747.5 **±** 211.5	**3.0·10^-4^**	157.2 (74.0 – 590.3)
Immunosuppression					
Induction therapy, n (%)					
Antithymocyte globulins	56 (37.8)	27 (33.8)	29 (42.6)	0.309	1.5 (0.7 – 3.0)
Non-depleting induction	72 (48.6)	42 (52.5)	30 (44.1)	0.327	0.7 (0.4 – 1.4)
No induction treatment	20 (13.5)	11 (13.7)	9 (13.3)	1	0.9 (0.4 – 2.5)
Maintenance therapy, n (%)					
CsA/Tac-Evl/Srl-S	103 (69.6)	57 (71.3)	46 (67.6)	0.721	0.8 (0.4 – 1.8)
CsA/Tac-MMF-S	20 (13.5)	10 (12.5)	10 (14.7)	0.811	1.2 (0.4 – 3.5)
Evl/Srl	19 (12.8)	10 (12.5)	9 (13.2)	1	1.1 (0.4 – 3.1)
Other	6 (4.1)	3 (3.8)	3 (4.4)	1	1.2 (0.2 – 9.1)
Transplantation outcome					
Delayed graft function, days (mean ± SD)	1.5 ± 3.6	1.5 ± 3.4	1.4 ± 4.1	0.871	-0.1 (-1.3; 1.1)
*De Novo* DSA, n (%)					
HLA Class I antibodies (MFI > 2500)	4 (2.7)	0	4 (5.9)	**0.042**	0.0 (0.0 – 1.3)
HLA Class II antibodies (MFI > 2500)	11 (7.4)	1 (0.0)	10 (14.7)	**0.003**	13.6 (1.7 – 109.6)
HLA Class I and II antibodies (MFI > 2500)	3 (2.0)	0	3 (4.4)	0.095	0.0 (0.0 – 2.0)
MICA antibodies (MFI > 1000)	4 (2.7)	0	4 (5.9)	**0.042**	0.0 (0.0 – 1.3)
One-year graft survival, n (%)	2 (1.4)	0	2 (2.9)	0.209	0.0 (0.0 – 4.5)
eGFR^b^ at 1 year, (mean ± SD)	65.26 **±** 23.27	77.50 **±** 17.51	49.19 **±** 19.76	**2.2·10^-16^**	-28.3 (-34.4; -22.3)
Serum creatinine^c^ at 1 year (mean ± SD)	112.4 **±** 59.3	86.2 **±** 17.6	144.3 **±** 74.8	**3.5·10^-10^**	58.1 (41.1 – 75.1)
eGFR^b^ at 3 years, (mean ± SD)	65.34 **±** 26.74	81.02 **±** 21.19	46.82 **±** 20.05	**< 2.2·10^-16^**	-34.2 (-40.9; -27.5)
Serum creatinine at 3 years (mean ± SD)	119.1 **±** 111.8	81.0 **±** 18.5	175.1 **±** 151.4	**1.5·10^-7^**	94.1 (60.4 – 127.8)
eGFR^b^ at 6 years, (mean ± SD)	59.15 ± 31.31	79.29 ± 19.05	31.26 ± 22.18	**< 2.2·10^-16^**	-48.0 (-54.7; -41.3)
Serum creatinine at 6 years (mean ± SD)	169.9 ± 188.3	86.2 **±** 32.6	291.0 **±** 247.3	**1.4·10^-11^**	204.8 (149.6 – 260.0)

(a) Mean differences (for continuous variables): x2 (patients with antibody-mediated rejection) - x1 (patients with stable graft function). (b) eGFR, calculated with the Modification of Diet in Renal Disease formula. (c) Serum creatinine was reported in µmol/L. ABMR, Antibody-mediated rejection; CI, Confidence Interval; CsA, Cyclosporin A; DGF, Delayed Graft Function; Evl, Everolimus; GFR, Glomerular Filtration Rate; IQR, Interquartile range; MMF, Mycophenolate mofetil; OR, Odds ratio; PRA, Panel-reactive antibody; S, Corticosteroids; SGF, Stable Graft Function; Srl, Sirolimus; TAC, Tacrolimus. All patients underwent their first kidney transplant and were negative for donor-specific antibodies (DSA) pre-transplantation.

Bold values denote statistical significance at the p < 0.05 level.

After the transplant, only 1.3% (1/80) of patients with stable graft function (SGF) developed *de novo* donor-specific antibodies (DSAs) with a MFI above 2500 for HLA or 1000 for MICA, compared to 17.6% (12/68) of patients with antibody-mediated rejection (ABMR) (OR = 16.9, 95% CI 2.1–134.0; P = 0.001). Most of these DSAs were directed against HLA class II antigens.

Additionally, 17 out of 68 (25.0%) patients with ABMR had MICA DSA alloantibodies (**Supplementary Table S3**). However, most of these patients had a low MFI level (<1000). Only 4 of the ABMR patients developed MICA DSAs with a mean fluorescence intensity (MFI) > 1000 (ranging from 1100 to 4500). Three of these patients had anti-MICA alloantibodies against the donor MICA-129 Methionine antigen (MICA 18 and MICA 01), while one patient had antibodies against both MICA-129 Methionine and Valine antigens (MICA 01, 08). Overall, 5 out of 6 anti-MICA DSAs with an MFI level > 1000 targeted Methionine at residue 129 (**Supplementary Table S3**).

Considerable overlap in treatment regimens administered before and after transplantation was observed between the two groups (**Table 2**). It is noteworthy to observe that the maintenance regimen based on mTOR inhibitors was statistically significantly associated with a higher number of episodes of ABMR compared to therapeutic regimens consisting of CNI [55.8% (48/86) vs. 32.3% (20/62); P = 0.007; OR: 2.6 (1.3 – 5.6)].

### 3.3 *MICA* allele frequencies and recipient/donor MICA alleles matching

*MICA* alleles were compared in 148 KTPs and 146 healthy controls. The analysis revealed a few substantial differences in allele frequencies between patients and healthy controls (**Table 3**). The frequency of the MICA*002:01 was significantly lower in the kidney transplant patient group compared to the control group [16.9% (50/296) in KTPs vs. 24.0% (70/292) in controls; p-value: 0.040; OR: 0.65 (0.42 - 0.99)]. The other alleles did not exhibit substantial differences and had comparable frequencies between controls and patients.

**Table 3:** *MICA* alleles their frequencies in the control population and kidney-transplant patients.

MICA Alleles	Control Population (N=146)		Kidney Transplant patients (N=148)			
	2N=292	%	2N=296	%	Odds Ratio	p-value^§^
001:01	42	0.144	50	0.169	1.209 (0.756-1.943)	0.428
002:01	70	0.240	50	0.169	0.645 (0.420-0.986)	**0.040**
004:01	28	0.096	34	0.115	1.223 (0.698-2.159)	0.503
007:01	10	0.034	12	0.041	1.191 (0.463-3.132)	0.829
008:01	42	0.144	36	0.122	0.824 (0.495-1.366)	0.467
008:02	1	0.003	3	0.010	2.975 (0.237-156.819)	0.624
008:04	2	0.007	5	0.017	2.488 (0.403-26.334)	0.450
009:01	22	0.075	20	0.068	0.890 (0.449-1.752)	0.751
009:02	6	0.021	2	0.007	0.325 (0.032-1.836)	0.174
010:01	4	0.014	9	0.030	2.255 (0.621-10.136)	0.262
011:01	14	0.048	14	0.047	0.986 (0.427-2.278)	1
012:01	4	0.014	8	0.027	1.998 (0.528-9.168)	0.383
012:02	1	0.003	3	0.010	2.975 (0.237-156.819)	0.624
016:01	14	0.048	18	0.061	1.285 (0.591-2.852)	0.586
017:01	8	0.027	8	0.027	0.986 (0.318-3.060)	1
018:01	20	0.068	19	0.064	0.933 (0.487-1.787)	0.870
019:01	2	0.007	2	0.007	0.986 (0.071-13.688)	1
027:01	2	0.007	3	0.010	1.484 (0.169-17.878)	1

^§^P-values were calculated for comparisons between control population group and Kidney Transplant patients. OR, odds ratio; CI, confidence interval; %, allele frequencies expressed as decimals. Bold formatting highlights values that have achieved statistical significance based on the applied tests or criteria.

Furthermore, when dividing the patients based on transplant outcomes (ABMR or SGF), the most represented alleles in both groups were MICA*001:01 and MICA*002:01 (**Table 4**). The MICA*010:01 allele was more frequent in SGF patients [0.007% (1/136) in ABMR, 0.05% (8/160) in SGF; P = 0.042; OR: 7.105 (0.877-57.548)]. Furthermore, ABMR and SGF groups were compared based on each allele’s heterozygote and homozygote frequencies. In particular, no significant differences were observed in the frequencies of homozygotes and heterozygotes for the most frequent allele: MICA*001:01, *002:01, *004:01, *008:01, *009:01 and *010:01 (**Table 4**).

**Table 4:** *MICA* alleles their frequencies in the group of antibody-mediated rejection patients and patients with stable graft function.

MICA Alleles	ABMR		SGF			
	2N=136	%	2N=160	%	Odds Ratio	p-value^§^
***001:01***	22	0.162	28	0.175	1.099 (0.571-2.137)	0.877
Hom	2	0.015	4	0.025	1.715 (0.241-19.230)	0.691
Het	18	0.132	20	0.125	0.937 (0.447-1.974)	0.863
***002:01***	20	0.147	30	0.188	1.337 (0.692-2.630)	0.437
Hom	2	0.015	6	0.038	2.603 (0.456-26.797)	0.296
Het	8	0.059	18	0.113	2.024 (0.804-5.571)	0.148
***004:01***	20	0.147	14	0.088	0.557 (0.249-1.217)	0.143
Hom	4	0.029	0	0.000	0.000 (0.000-1.275)	**0.044**
Het	12	0.088	14	0.088	0.991 (0.408-2.440)	1
***007:01***	4	0.029	8	0.050	1.734 (0.452-8.049)	0.556
Hom	0	0.000	0	0.000	–	1
Het	4	0.029	8	0.050	1.734 (0.452-8.049)	0.556
***008:01***	18	0.132	18	0.113	0.832 (0.389-1.779)	0.722
Hom	2	0.015	0	0.000	0.000 (0.000-4.518)	0.210
Het	14	0.103	18	0.113	1.104 (0.495-2.508)	0.852
***008:02***	1	0.007	2	0.013	1.706 (0.088-101.495)	1
Hom	0	0.000	0	0.000	–	1
Het	1	0.007	2	0.013	1.706 (0.088-101.495)	1
***008:04***	4	0.029	1	0.006	0.209 (0.004-2.141)	0.184
Hom	0	0.000	0	0.000	–	1
Het	4	0.029	1	0.006	0.209 (0.004-2.141)	0.184
***009:01***	6	0.044	14	0.088	2.073 (0.722-6.781)	0.167
Hom	0	0.000	0	0.000	–	1
Het	6	0.044	14	0.088	2.073 (0.722-6.781)	0.167
***009:02***	1	0.007	1	0.006	0.850 (0.011-67.103)	1
Hom	0	0.000	0	0.000	–	1
Het	1	0.007	1	0.006	0.850 (0.011-67.103)	1
***010:01***	1	0.007	8	0.050	7.070 (0.928-317.112)	**0.042**
Hom	0	0.000	0	0.000	–	1
Het	1	0.007	8	0.050	7.070 (0.928-317.112)	**0.042**
***011:01***	8	0.059	6	0.038	0.624 (0.174-2.113)	0.422
Hom	0	0.000	0	0.000	–	–
Het	8	0.059	6	0.038	0.624 (0.174-2.113)	0.422
***012:01***	4	0.029	4	0.025	0.847 (0.155-4.638)	1
Hom	0	0.000	0	0.000	–	1
Het	4	0.029	4	0.025	0.847 (0.155-4.638)	1
***012:02***	1	0.007	2	0.013	1.706 (0.088-101.495)	1
Hom	0	0.000	0	0.000	–	1
Het	1	0.007	2	0.013	1.706 (0.088-101.495)	1
***016:01***	8	0.059	10	0.063	1.066 (0.367-3.210)	1
Hom	0	0.000	2	0.013	–	0.502
Het	8	0.059	6	0.038	0.624 (0.174-2.113)	0.422
***017:01***	4	0.029	4	0.025	0.847 (0.155-4.638)	1
Hom	0	0.000	0	0.000	–	1
Het	4	0.029	4	0.025	0.847 (0.155-4.638)	1
***018:01***	11	0.08	8	0.05	1.672 (0.653-4.284)	0.344
Hom	0	0.000	0	0.000	–	1
Het	11	0.081	8	0.050	0.599 (0.203-1.693)	0.344
***019:01***	1	0.007	1	0.006	0.850 (0.011-67.103)	1
Hom	0	0.000	0	0.000	–	1
Het	1	0.007	1	0.006	0.850 (0.011-67.103)	1
***027:01***	2	0.015	1	0.006	0.423 (0.007-8.201)	0.598
Hom	0	0.000	0	0.000	–	1
Het	2	0.015	1	0.006	0.423 (0.007-8.201)	0.598

ABMR, antibody-mediated rejection; SGF, Stable Graft Function; § p-values were calculated for comparisons between ABMR and SGF groups. OR, odds ratio; CI, confidence interval; % = allele frequencies expressed as decimals. Bold values mean statistical significance. Bold formatting highlights values that have achieved statistical significance based on the applied tests or criteria.

**Figure 2:**
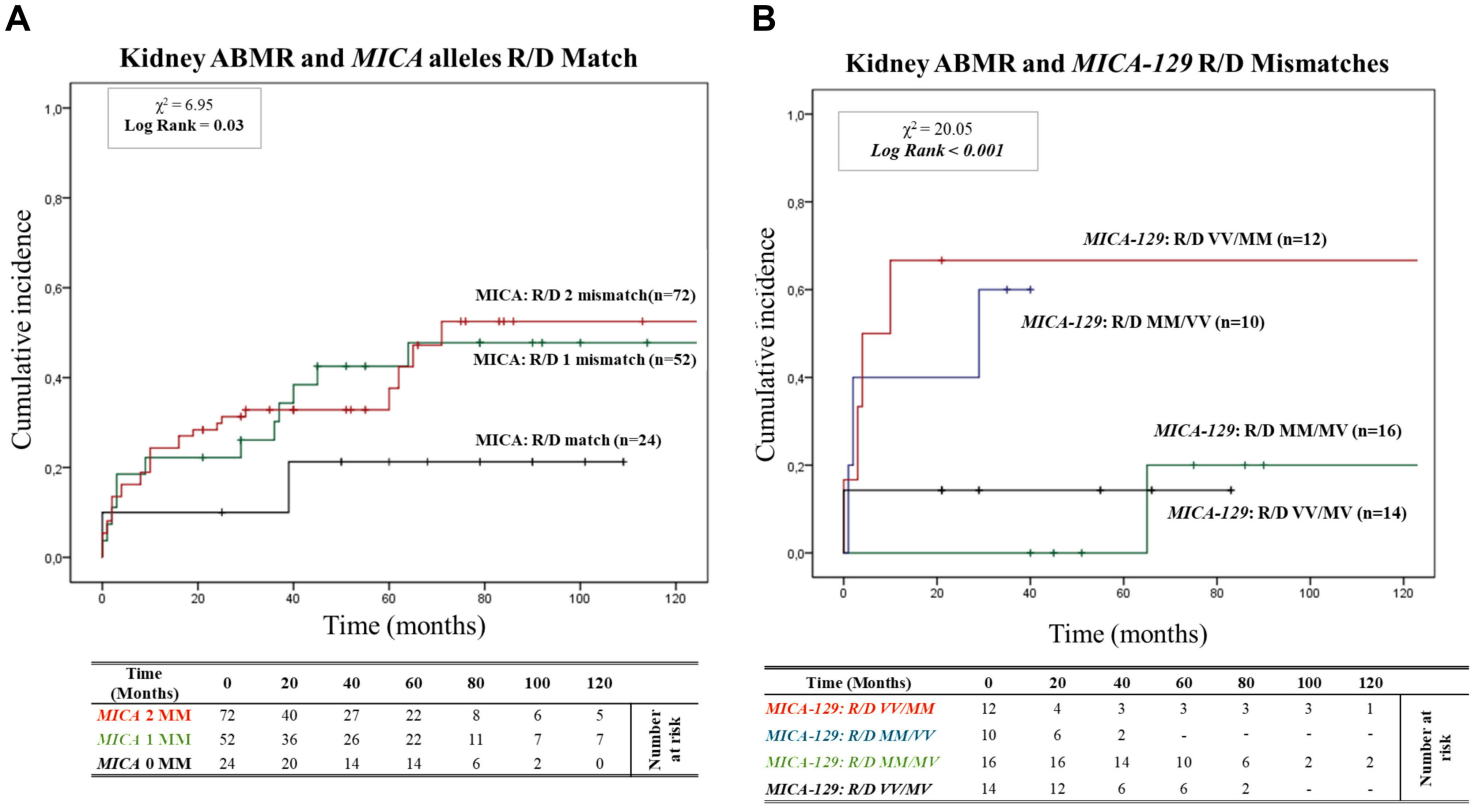
**(A)** Cumulative incidence for antibody-mediated rejection according to recipient-donor (R/D) *MICA* allele mismatches. The cumulative incidence of rejection events is graphically presented for a cohort of 148 patients observed over 120 months. Patients were categorized based on three groups of patients stratified according to donor-recipient *MICA* allele mismatches [0MM (black), 1MM (green), 2 MM (red)]. P-values were calculated using the two-sided Log-rank test without correction. χ2: Chi-square. MM: Mismatches. **(B)** Cumulative incidence for antibody-mediated rejection according to recipient-donor (R/D) MICA-129 allele mismatches. The cumulative incidence of rejection events is graphically presented for a cohort of 148 patients observed over 120 months. Patients were categorized on four groups of patients stratified according to recipient-donor MICA allele mismatches based on substitution of valine (V) with methionine (M) at position 129 (MICA-129) of the MICA protein: [R/D VV/MM (Red), R/D MM/VV (blue), R/D MM/MV (green) and R/D VV/MV (black)]. P-values were calculated using the two-sided Log-rank test without correction. χ2, Chi-square.

**Figure 2A** shows the 95% cumulative incidence curves over 120 months for antibody-mediated rejection (ABMR) in 148 patients, stratified into three groups based on the number of *MICA* allele mismatches with their donors. Of these, 24 R/D pairs (16.2%) were matched (0 MM), 52 R/D pairs (35.1%) had one mismatch (1 MM), and 72 R/D pairs (48.7%) had two mismatches (2 MM). The median follow-up was 52.9 months for MICA-matched patients and 64.7 months for MICA-mismatched patients. At 5 years post-transplantation, graft survival was 79.2% (19/24) for *MICA*-matched patients and 64.5% (80/124) for *MICA*-mismatched patients (1 and 2 MM). Compared to MICA-mismatched patients, those matched for *MICA* alleles exhibited a significantly reduced risk of antibody-mediated rejection (X² = 6.95; Log-rank = 0.03). Indeed at 120 months post-transplantation, the incidence of ABMR was only 20.8% (5/24) in *MICA*-matched patients, compared to 49.1% (26/52) and 52.7% (38/72) in patients with 1 and 2 mismatches (1 MM and 2 MM), respectively.

In addition to a detailed exploration of the consequences of the *MICA* mismatching model, we closely monitored renal function through assessments of serum creatinine levels and glomerular filtration rate (eGFR) (**Supplementary Figures S2**, **S3**). This additional analysis further confirmed the impact of recipient/donor (R/D) MICA allele matching on renal outcomes.

Building on this analysis, we further investigated the specific role of MICA-129 mismatches and their potential interactions with antibody-mediated rejection. We analyzed the cumulative incidence curves over 120 months for ABMR based on the R/D MICA-129 1 or 2 mismatches (1 MM and 2 MM) as R/D: MM/MV, VV/MV, MM/VV, VV/MM (**Figure 2B**). Two MICA-129 mismatches were observed in 12 R/D pairs VV/MM (8.1%) and 10 R/D pairs MM/VV (6.8%), while 1 MICA-129 mismatch was observed in 16 R/D pairs MM/MV (10.8%) and 14 R/D pairs VV/MV (9.5%).

The median follow-up was 27.9 months for MICA-129 2 mismatched patients (VV/MM and MM/VV) and 60.6 months for patients with 1 MICA-129 mismatch compared to the donor (VV/MV and MM/MV). Patients with 2 MICA-129 mismatches exhibited a significantly higher risk of antibody-mediated rejection (X² = 20.05; Log-rank < 0.001).

At 5 years post-transplantation, graft survival was 100% (16/16) for MICA-129 1 mismatch R/D MM/MV, and 85.7% (12/14) for MICA-129 1 mismatch R/D VV/MV, while in the presence of 2 MICA-129 mismatches, graft survival decreased to 40% (4/10) for R/D MM/VV pairs and to 33.3% (4/12) for R/D VV/MM pairs.

At 120 months post-transplantation, the cumulative incidence of ABMR remained significantly higher in the pairs that presented two MICA-129 mismatches, particularly in R/D VV/MM pairs [66.7% (8/12)].

### 3.4 Association of recipient *_R_NKG2D rs1049174 (G>C)* and *rs2255336 (A>G)* polymorphisms and antibody-mediated rejection risk

#### 3.4.1 *_R_NKG2D rs1049174 (G>C)* and *rs2255336 (A>G)* Hardy–Weinberg equilibrium and linkage disequilibrium analysis

The two index SNPs, *rs1049174 (G>C)* and *rs2255336 (A>G)*, located in the _R_NKG2D gene, were analyzed in 146 healthy control individuals and 148 kidney transplant patients stratified into two groups: SGF and ABMR. The comparison did not reveal significant differences in the frequencies of these SNPs. (**Tables 5A, B**). The *rs1049174* (G>C) variant was found to be in Hardy–Weinberg equilibrium (HWE) within the control group population and the Stable graft function (SGF) group [X^2HWE^ = 0.1222; p = 0.726661 and X^2HWE^ = 0.8765; p = 0.34916 respectively] (**Tables 5A, B**). Only patients with ABMR had frequencies deviating from HW expectations [X^2HWE^ = 11.0717; p= 0.000877] (**Table 5B**). Consequently, the entire group of kidney transplant patients lost the HWE [X^2HWE^ = 9.0317; p = 0.002653] (**Table 5A**). In contrast, *rs2255336 (A>G)* was in HWE within the control population group and kidney transplant patients [X^2HWE^ = 1.828; p = 0.1763 and X^2HWE^ = 0.0587; p = 0.8080 respectively] (**Table 5A**) and the due subgroup ABMR and SGF [X^2HWE^ = 0.2369; p = 0.626 and X^2HWE^ = 0.7405; p = 0.3895 respectively] as shown in detail in (**Table 5B**). Moreover, these two SNPs in the *NKG2D* gene, despite being a few thousand bases apart (21), do not show strong linkage disequilibrium in all examined groups (**Supplementary Tables S2A–C**).The only observed association pertains to the SNP*s rs1049174G* with *rs2255336A*, which exhibit weak LD in the patient group (*D’=* 0.70, *r* ^2^ = 0.32; *X* ^2^ = 14.87, P =0.0001) including the two subgroups SGF and CR (*D’=* 0.74, *r* ^2^ = 0.41; *X* ^2^ = 9.80, P =0.002 and *D’=* 0.66, *r* ^2^ = 0.24; *X* ^2^ = 4.59, P =0.032, respectively).

**Table 5A:** Allele and Genotype distribution of *rs1049174* and *rs2255336* in the control population and kidney-transplant patients.

Allele and Genotype distribution						
Gene	SNP	Control population (n=146)			Kidney transplant patients (n=148)	
*NKG2D*	*rs1049174* (G>C)	Allele	(%)	*X^2^* HWE	(%)	*X^2^* HWE
		G	0.6615	0.1222,	0.412	9.0317,
		C	0.3385	p = 0.726661	0.588	p= **0.002653**
		Genotype	(%)		(%)	
		GG	16 (0.108)		34 (0.230)	
		GC	67 (0.462)		54 (0.368)	
		CC	63 (0.431)		60 (0.405)	
*NKG2D*	*rs2255336* (A>G)	Allele	(%)	*X^2^* HWE	(%)	*X^2^* HWE
		A	0.2911	1.8284,	0.3108	0.0587,
		G	0,7089	p = 0.1763	0.6892	p = 0.808
		Genotype	(%)		(%)	
		AA	9 (0.0616)		14 (0.095)	
		AG	67 (0.462)		61 (0.405)	
		GG	70 (0.479)		73 (0.493)	

*X*2HWE, Hardy-Weinberg equilibrium Chi square value; *p*, Hardy-Weinberg equilibrium p value; %, allele frequencies expressed as decimals.

Bold values denote statistical significance at the p < 0.05 level.

**Table 5B:** Allele and Genotype distribution of *rs1049174* and *rs2255336* in the kidney-transplant patients.

Allele and Genotype distribution						
Gene	SNP	SGF (n=80)			ABMR (n=68)	
*NKG2D*	*rs1049174* (G>C)	Allele	(%)	*X^2^* HWE	(%)	*X^2^* HWE
		G	0.3875	0.8765,	0.4412	11.0717,
		C	0.6125	p = 0.349162	0.5588	p = **0.000877**
		Genotype	(%)		(%)	
		GG	14 (0.175)		20 (0.294)	
		GC	34 (0.425)		20 (0.294)	
		CC	32 (0.400)		28 (0.412)	
*NKG2D*	*rs2255336* (A>G)	Allele	(%)	*X^2^* HWE	(%)	*X^2^* HWE
		A	0.3125	0.2369,	0.3088	0.7405,
		G	0.6875	p = 0.626	0.6912	*p* = 0.389501
		Genotype	(%)		(%)	
		AA	6 (0.075)		8 (0.118)	
		AG	35 (0.438)		26 (0.382)	
		GG	39 (0.488)		34 (0.500)	

*X*2HWE, Hardy-Weinberg equilibrium Chi square value; *p*, Hardy-Weinberg equilibrium p value; %, allele frequencies expressed as decimals. ABMR, antibody-mediated rejection; SGF, Stable.

Bold values denote statistical significance at the p < 0.05 level.

#### 3.4.2 Association of *_R_NKG2D rs1049174 (G>C)* polymorphisms and antibody-mediated rejection risk

**Figure 3:**
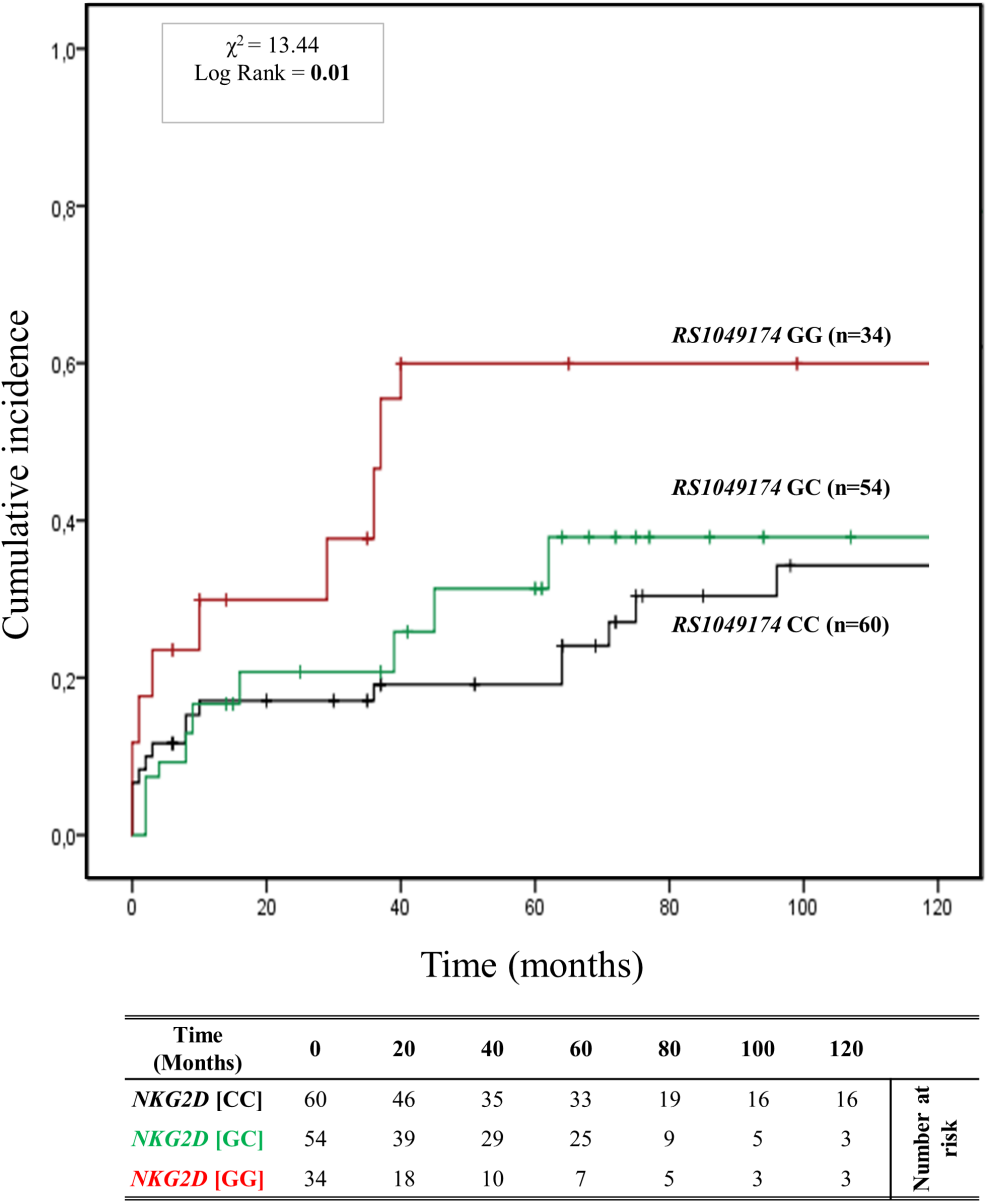
Cumulative incidence for antibody-mediated rejection according to *NKG2D rs1049174 (G>C)* genotype. The cumulative incidence of rejection events is graphically presented for a cohort of 148 patients observed over 120 months. Patients were categorized into three groups based on their *NKG2D* genotype for *rs1049174 (G>C)* [GG (red), GC (green), CC (black)]. This allele is linked to the haplotype blocks *NKG2D* hb-1, which produces NKG2D_R_ with low (*rs1049174* [CC]; LNK) or high (*rs1049174* [GG]; HNK) natural cytotoxic activity phenotypes. P-values were calculated using the two-sided Log-rank test without correction. χ2, Chi-square.

**Figure 3** depicts the cumulative incidence of ABMR over 120 months in patients categorized based on the three genotypes of *rs1049174* (G>C) in the *NKG2D* gene (GG, CG, and CC). Thirty-four patients (23%) had the *rs1049174* [*GG*] genotype, 54 (36.5%) were heterozygous [CG], and 60 (40.5%) were homozygous [CC]. At 5 years post-transplantation, graft survival was only 38.2% (13/34) for patients with the *rs1049174* [GG] genotype, compared to 70.4% (38/54) and 91.7% (49/60) for patients with the *rs1049174* [CG] and *rs1049174* [CC] genotypes, respectively.

Patients with the *rs1049174* [GG] genotype exhibited a significantly increased risk of antibody-mediated rejection (X² = 13.44; Log-rank = 0.001). Indeed, at 120 months post-transplantation, the cumulative incidence of ABMR was higher (61.8% (21/34)) in the group of patients with the *rs1049174* [GG] genotype compared to 42.6% (23/54) and 40.0% (24/60) in patients with the *rs1049174* [CG] and *rs1049174* [CC] genotypes, respectively.

This evidence was also confirmed by the trends in renal function, as assessed through serum creatinine levels (SCr) and glomerular filtration rate (eGFR). Difference in eGFR was already evident at 12 months [(57.90 ± 25.98 mL/min/1.73 m^2^ [GG] vs 63.34 ± 22.74 mL/min/1.73 m^2^ [CG] vs 71.22 ± 21.93 mL/min/1.73 m^2^ [CC]; P = 0.085], at 36 months [(52.42 ± 23.22 mL/min/1.73 m^2^ [GG] vs 58.23 ± 26.92 mL/min/1.73 m^2^ [CG] vs 77.60 ± 24.74 mL/min/1.73 m^2^ [CC]; P = 0.007], and continued to rise at 72 months [(42.60 ± 27.43 mL/min/1.73 m^2^ [GG] vs 63.18 ± 30.48 mL/min/1.73 m^2^[CG] vs 64.02 ± 31.50 mL/min/1.73 m^2^ [CC]; P = 0.037] after transplantation. The P_AUC_ was also statistically significant (P_AUC_ = 0.002), (**Supplementary Figure S4**).

Concurrently, in patients with the r*s1049174* [GG] genotype, mean SCr levels were worse than those of patients with the other two genotypes, *rs1049174* [CG] and *rs1049174* [CC]. In fact, they were significantly higher at 12 months [(128.65 ± 64.52 μmol/L [GG] vs 121.20 ± 73.37 μmol/L [CG] vs 99.28 ± 28.67 μmol/L [CC]; P = 0.046], at 36 months [(140.14 ± 65.93 μmol/L [GG] vs 122.52 ± 57.24 μmol/L [CG] vs 92.55 ± 35.97 μmol/L [CC]; P = 0.009], and continued to increase at 72 months [(195.58 ± 121.70 μmol/L [GG] vs 137.80 ± 168.75 μmol/L [CC] vs 125.87 ± 89.65 μmol/L [CC]; P = 0.042]. Similarly to the eGFR curves, the P_AUC_ for mean SCr levels was also statistically significant (P_AUC_ = 0.023), (**Supplementary Figure S5**). Moreover, it is noteworthy that the influence exerted by the *NKG2D rs1049174 GG* polymorphism on the transplant outcome remains independent of other clinical and genetic variables, as elucidated by the multivariate logistic regression analysis (**Supplementary Table S4**).

#### 3.4.3 Association of *_R_NKG2D rs2255336 (A>G)* polymorphisms and antibody-mediated rejection risk

**Figure 4:**
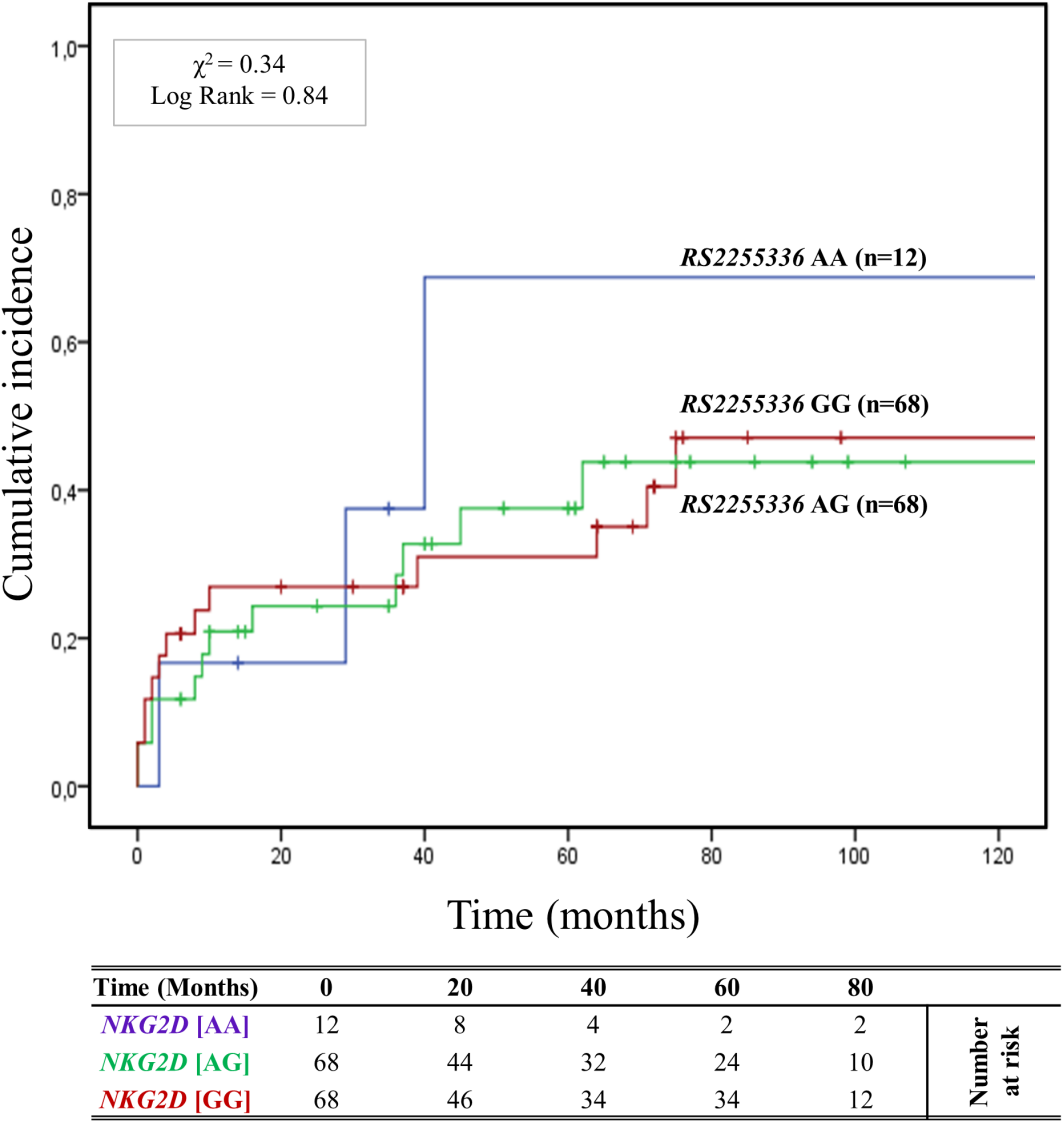
Cumulative incidence for antibody-mediated rejection according to *NKG2D rs2255336 (A>G)* genotype. The cumulative incidence of rejection events is graphically presented for a cohort of 148 patients observed over 120 months. Patients were categorized into three groups based on their *NKG2D* genotype for *rs2255336 (A>G)* [*AA (light blue), AG (green), GG (red)].* This allele is linked to the haplotype blocks NKG2D hb-2, which produces *_R_* NKG2D with low (*rs2255336* [GG]; LNK) or high (*rs2255336* [AA]; HNK) natural cytotoxic activity phenotypes. P-values were calculated using the two-sided Log-rank test without correction. χ2, Chi-square.

The cumulative incidence over 120 months of antibody-mediated rejection (ABMR) in patients divided based on the three genotypes (AA, AG, and GG) of the other haploblock *NKG2D* identified by the *rs2255336* (A>G) as highlighted in **Figure 4**. Twelve (8.2%) patients had the *rs2255336* [AA] genotype, 68 (45.9%) were heterozygous [*AG*], and 68 (45.9%) were homozygous *[GG]*. At 5 years post-transplantation, graft survival was only 33.3% (4/12) for patients with the *rs2255336 [AA*] genotype, compared to 63.9% (43/68) and 69.1% (47/68) for patients with the rs2255336 [AG] and *rs2255336 [GG]* genotypes, respectively. Patients with the *rs2255336* [AA] genotype exhibited a significantly increased risk of antibody-mediated rejection. At 120 months after transplantation, the incidence of antibody-mediated rejection was 66.7% (8/12) in these patients, while in patients with *rs2255336* [AG] and *rs2255336* [GG] genotypes, it was 41.2% (28/68) and 47.1% (32/68), respectively. However, the Mantel-Cox log-rank test did not reach statistical significance (*X* ^2^ = 0.34; Log-rank = 0.84). Interestingly, this polymorphism appears to influence the eGFR of transplanted patients over time. Individuals with the *rs2255336* [AA] genotype exhibited lower eGFR values compared to patients with the other two genotypes, *rs2255336* [AG] and *rs2255336* [GG], already at 12 months [(55.60 ± 32.13 mL/min/1.73 m^2^ [AA] vs 63.40 ± 23.58 mL/min/1.73 m^2^[AG] vs 69.41 ± 21.31 mL/min/1.73 m^2^ [GG]; P = 0.194]. This difference reached statistical significance at 36 months [(39.31 ± 20.38 ml/min [AA] vs 60.45 ± 28.05 mL/min/1.73 m^2^ (AG) vs 73.53 ± 24.29 mL/min/1.73 m^2^ [GG]; P = 0.047] and continued to rise at 72 months [(26.34 ± 20.32 mL/min/1.73 m^2^ [GG] vs 62.10 ± 31.89 mL/min/1.73 m^2^ [AG] vs 61.45 ± 29.28 mL/min/1.73 m^2^ [GG]; P = 0.015] after transplantation. In this case, the P_AUC_ also reached statistical significance (P_AUC_ = 0.028), (**Supplementary Figure S6**). SCr levels also appear to be significantly influenced by the three genotypes of _R_ *NKG2D rs2255336* (P_AUC_ = 0.030), (**Supplementary Figure S7**). Higher values are observed in the presence of the *rs2255336* [AA] genotype and tend to progressively increase over time: at 12 months [(154.13 ± 89.45 μmol/L [AA] vs 119.19 ± 67.38 μmol/L [AG] vs 100.31 ± 27.70 μmol/L [GG]; P = 0.073], at 36 months [(189.38 ± 68.47 μmol/L [AA] vs 148.80 ± 154.93 μmol/L [AG] vs 95.95 ± 35.32 μmol/L [GG]; P = 0.307] and continued to increase at 72 months [(286.55 ± 154.16 μmol/L [AA] vs 170.28 ± 216.80 μmol/L [AG] vs 152.21 ± 165.73 μmol/L [GG]; P = 0.160].

### 3.5 Combined effect of R/D *MICA* allele mismatch and *_R_NKG2D* genotypes of *rs1049174 (G>C)* on kidney transplantation

**Figure 5:**
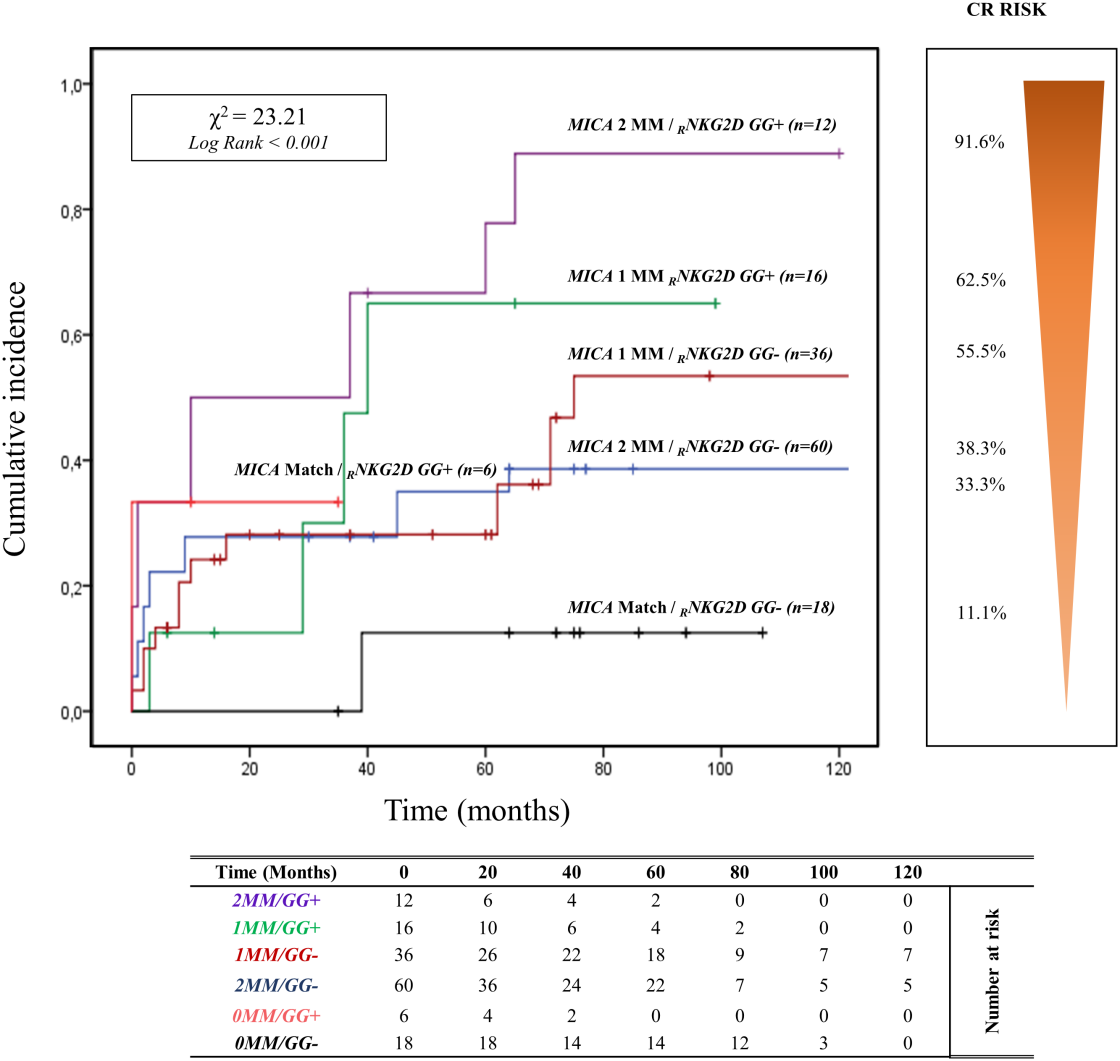
Cumulative incidence for antibody-mediated rejection according to *NKG2D rs1049174 [GG]* genotype and *MICA* allele mismatches. The cumulative incidence of rejection events is graphically presented for a cohort of 148 patients observed over 120 months. Patients were categorized based on their *NKG2D* genotype GG for and *rs1049174* in combination with the donor-recipient *MICA* allele mismatches. The *rs1049174* [GG], which produces *_R_NKG2D* with high natural cytotoxic activity phenotypes, has been correlated with donor-recipient *MICA* allele mismatches. Six groups were formed based on the number of MICA allele mismatches and the presence or absence of the GG (*rs1049174)* genotype: 1. Two *MICA* R/D allele mismatches and *rs1049174 _R_NKG2D[GG] (purple)* 2. One *MICA* R/D allele mismatches and *rs1049174 _R_NKG2D[GG] (green)* 3. One *MICA* R/D allele mismatches and *rs1049174 _R_NKG2D[GG] (red)* 4. Two *MICA* R/D allele mismatch and *rs1049174 _R_NKG2D[CG]* and *[CC] (light blue)* 5. *MICA* R/D alleles match and *rs1049174 _R_NKG2D[GG] (orange)* 6. *MICA* R/D alleles match and *rs1049174 _R_NKG2D[CG]* and *[CC] (black).* P-values were calculated using the two-sided Log-rank test without correction. χ2, Chi-square. MM, mismatches.

The curve of cumulative incidence over 120 months of ABMR highlights the effect of different combinations of R/D *MICA* allele mismatch with the three genotypes of *rs1049174* (G>C) in the *NKG2D* gene (GG, CG, and CC). The six curves (**Figure 5**) are well distinct and show a gradient of ABMR risk: 2MM/GG+ (91.6%, 11/12) > 1MM/GG+ (62.5%, 10/16) > lMM/GG- (55.5%, 20/36) > 2MM/GG- (38.3%, 23/60) > 0MM/GG+ (33.3%, 2/6) > 0MM/GG- (11.1%, 2/18). Therefore, the highest risk of rejection occurs in the patients with the *rs1049174* GG+ genotype transplanted with a donor with complete *MICA* allele mismatch (2MM/GG+). Conversely, patients with rs1049174 GG- and *MICA* allele match with the donor (0MM/GG-) present a minimal incidence of ABMR (X^2^ = 23.21; Log-rank < 0.001).

Renal function, monitored through SCr levels and eGFR, appears to be closely influenced by the combination of R/D *MICA* allele mismatch with the three genotypes of *rs1049174* (G>C) in the *NKG2D* gene (**Supplementary Figures S8**, **S9** respectively). Moreover, the adverse effect exerted by the *2MM/GG+* combination on the kidney transplant outcome is observed in the subgroup of patients with *HLA-DRB1* and *HLA-DQB1* match with the donor and those with HLA II class mismatch.

**Figure 6:**
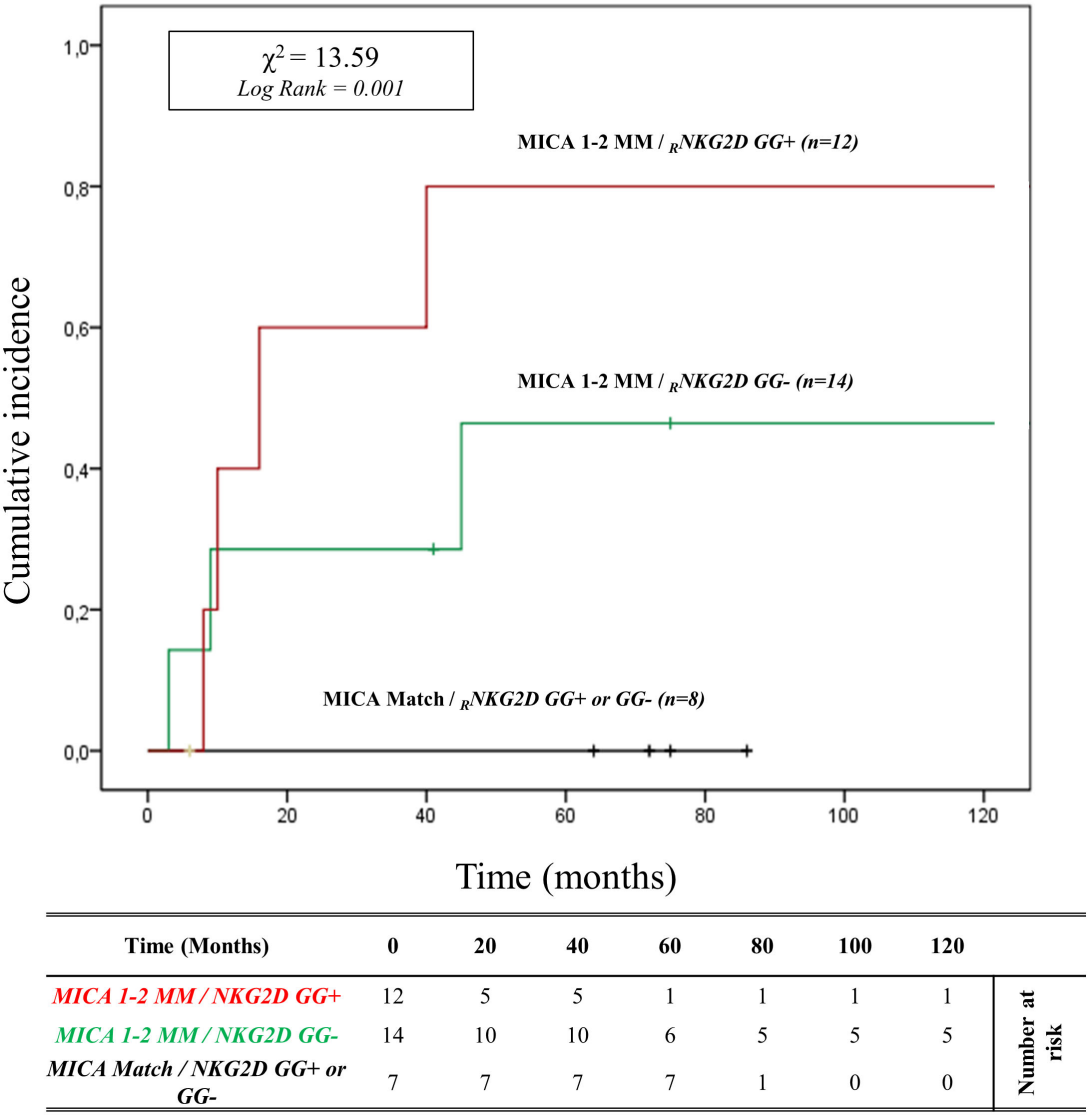
Cumulative incidence for antibody-mediated rejection in patient’s *HLA-DRB1* and *HLA-DQB1* match R/D according to *NKG2D* genotype and *MICA* allele mismatches. The cumulative incidence of rejection events is graphically presented for a cohort of 33 patients with *HLA-DRB1* and *HLA-DQB1* match R/D observed over 120 months. Patients were categorized into three groups based on the following criteria: 1. R/D *MICA* alleles match independently of the *NKG2D rs1049174* genotype (*black*). 2. R/D *MICA* alleles 1-2 mismatches with *NKG2D rs1049174 CG* or *CC* genotype (marked as GG-) *(green)*. 3. R/D *MICA* alleles 1-2 mismatch with *NKG2D rs1049174 GG* genotype *(red)*. P-values were calculated using the two-sided Log-rank test without correction. χ2, Chi-square. MM, mismatches; R/D, recipient-donor.

In patients with *HLA-DRB1* and *HLA-DQB1* full match, the cumulative incidence curve over 120 months illustrates that the highest risk of rejection occurs in individuals with the *2MM/GG+* and *1MM/GG+* combinations (83.3%, 10/12). Conversely, no patients with *0MM/GG+* and *0MM/GG-* (0%, 0/8) show episodes of ABMR (*X* ^2^ = 13.59; Log rank = 0.001; **Figure 6**). Similar results are observed when analyzing the remaining and more numerous subgroups of patients with 1 or 2 *HLA-DRB1* and *HLA-DQB1* allele mismatches with the donor (*X* ^2^ = 14.81; Log-rank = 0.002; **Supplementary Figure S10**).

**Figure 7:**
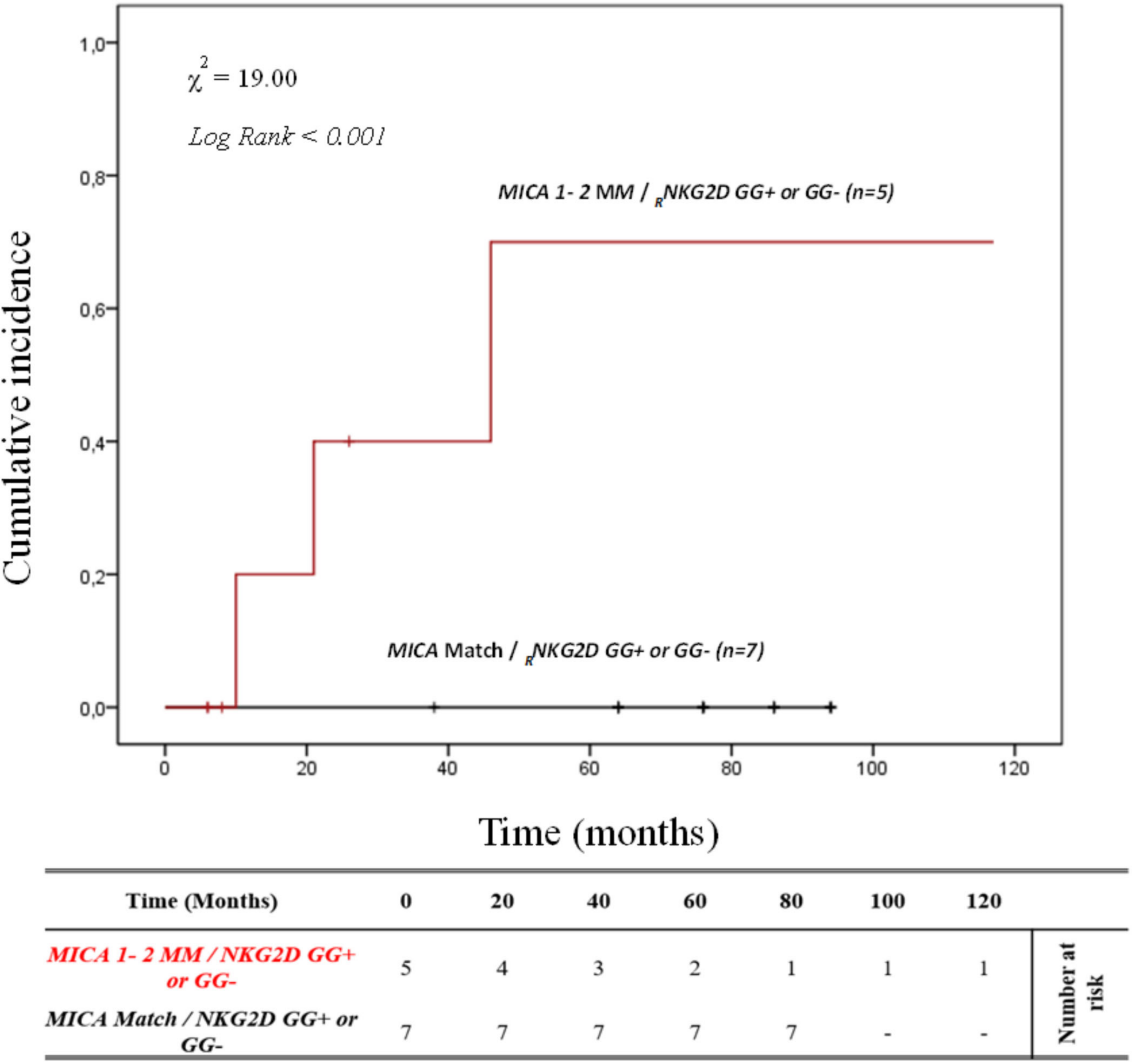
Cumulative incidence for antibody-mediated rejection in patient’s *HLA-B* match R/D according to *NKG2D* genotype and *MICA* allele mismatches. The cumulative incidence of rejection events is graphically presented for a cohort of 12 patients with *HLA-B* match R/D observed over 120 months. Patients were categorized into two groups based on the following criteria: 1. R/D *MICA* alleles match independently of the *NKG2D rs1049174* genotype (*black*). 2. R/D *MICA* alleles 1-2 mismatch independently of the *NKG2D rs1049174* genotype *(red)*. P-values were calculated using the two-sided Log-rank test without correction. χ2, Chi-square. MM, mismatches; R/D, recipient-donor.

It’s well known that there is linkage disequilibrium between *MICA* and *HLA-B* due to the proximity of these genes. To better determine the contribution of *MICA* allele mismatches in the development of ABMR, we analyzed the *HLA-B* full-match D/R pairs separately. In this small cohort of patients (n=12), the cumulative incidence curve over 120 months illustrates that the highest risk of rejection occurs in individuals with the *1-2MM/GG+* and *1-2MM/GG-* combinations (80.0%, 4/5). Conversely, no patients with *0MM/GG+* and *0MM/GG-* (0%, 0/7) show episodes of ABMR (*X* ^2^ = 19.00; Log rank < 0.001; **Figure 7**).

This data suggests that the effect of the different combinations of these polymorphisms is independent of *HLA* class I and class II matching.

Furthermore, the effect exerted by the *2MM/GG+* combination on the kidney transplant outcome is also independent of other clinical and genetic variables, as highlighted by the multivariate logistic regression analysis (**Supplementary Table S3**).

Finally, since the R/D MICA-129 mismatches were significant in the previous analysis, we examined it in combination with the *rs1049174* [*GG*] polymorphism of the *NKG2D* receptor (**Supplementary Figure S1A**). The cumulative incidence curves over 120 months for ABMR based on R/D MICA-129 1 or 2 mismatches (1 MM and 2 MM) and the *NKG2D rs1049174* [*GG*] polymorphism, were categorized as R/D: MM/MV, VV/MV, MM/VV, and VV/MM (**Supplementary Figure S1A**). Two MICA-129 mismatches were identified in 8 R/D pairs with the VV/MM/GG+ genotype (15.3%) and 4 R/D pairs with the MM/VV/GG+ genotype (7.7%), while 10 patients with 2 mismatches did not carry the *NKG2D rs1049174* [GG] polymorphism (4 R/D pairs VV/MM/GG- (7.7%) and 6 MM/VV/GG- (11.5%). Among patients with one mismatch, there were 16 R/D pairs MM/MV/GG- (30.8%) and 14 R/D pairs VV/MV/GG- (26.9%). The median follow-up was only 15.3 months for patients with 2 MICA-129 mismatches and the presence of *NKG2D rs1049174* [GG] (VV/MM/GG+ and MM/VV/GG+), while it was 35.1 months for those with 2 MICA-129 mismatches and absence of *NKG2D rs1049174* [GG] (VV/MM/GG- and MM/VV/GG-). In contrast, in patients with one MICA-129 mismatch the median follow-up was 79.3 months for MM/MV/GG- and 60.6 months for VV/MV/GG-.

At 5 years post-transplantation, for patients with 1 MICA-129 mismatch and *rs1049174* [GG-], graft survival was 100% (16/16) for R/D pairs MM/MV/GG- and 85.7% (12/14) for R/D pairs VV/MV/GG-. In the presence of 2 MICA-129 mismatches and *rs1049174* [GG+], graft survival was 87.5% (7/8) for R/D pairs VV/MM/GG+ and 0% (0/4) for R/D pairs MM/VV/GG+. When 2 MICA-129 mismatches were associated with rs1049174 [GG-], 5-year graft survival was 50% (2/4) in R/D pairs VV/MM/GG- and 66.7% (4/6) for R/D pairs MM/VV/GG-.

Patients with 2 MICA-129 mismatches and the presence of NKG2D *rs1049174* [*GG*] showed a significantly higher risk of antibody-mediated rejection (X² = 27.33; Log-rank < 0.001).

Notably, at 120 months post-transplantation, the cumulative incidence of ABMR was 100% in patients with 2 MICA-129 mismatches combined with the rs1049174 [GG] genotype (MM/VV/GG+ and VV/MM/GG+). In contrast, in the absence of the *rs1049174* [GG] genotype (VV/MM/GG- and MM/VV/GG-), the cumulative incidence of rejection decreased to 50% (2/4) and 33.3% (2/6), respectively (**Supplementary Figure S1A**).

Lastly, we also analyzed cumulative incidence curves over 120 months for ABMR based on combination R/D MICA-129 1 or 2 mismatches (1 MM and 2 MM) and the other *NKG2D* polymorphism *(rs2255336* [AA]), categorized as R/D: MM/MV, VV/MV, MM/VV, and VV/MM (**Supplementary Figure S1B**). Two MICA-129 mismatches were identified in 2 R/D pairs with the MM/VV/AA+ genotype (3.8%), while 20 patients with 2 mismatches did not carry the *NKG2D rs2255336* [AA] polymorphism (12 R/D pairs VV/MM/AA- (23.1%) and 8 MM/VV/AA- (15.4%)). Among patients with one mismatch, there were 16 R/D pairs MM/MV/AA- (30.8%) and 14 R/D pairs VV/MV/AA- (26.9%). The median follow-up was 29.1 months for patients with 2 MICA-129 mismatches and the presence of *rs2255336* [AA] (MM/VV/AA+), while it was 27.8 months for those with 2 MICA-129 mismatches and absence of *rs2255336* [AA] (VV/MM/AA- and MM/VV/AA-). Instead, patients with only 1 MICA-129 mismatch and absence of *rs2255336* [AA] (VV/MV/AA- and MM/MV/AA-) had a longer follow-up period (60.6 months).

At 5 years post-transplantation, graft survival was 0% (0/2) for R/D pairs with 2 MICA-129 mismatches and *rs2255336* [AA+] (MM/VV/AA+), 33.3% (4/12) for R/D pairs with 2 MICA-129 mismatches and *rs2255336* [AA-] (VV/MM/AA-), and 50.0% (4/8) for MM/VV/AA-. Instead, patients with just one MICA-129 mismatch the graft survival was 100% (16/16) for MM/MV/AA- and 85.7% (12/14) VV/MV/AA-.

A significantly higher risk of antibody-mediated rejection was associated with the combination of 2 MICA-129 mismatches and the presence of *NKG2D rs2255336* [AA] (X² = 20.32; Log-rank < 0.001).

### 3.6 Correlation between maintenance therapy and the onset of ABMR based on the genetic profiles of kidney transplant recipients

**Table 6** highlights how the effectiveness of maintenance regimens in countering graft rejection is correlated with the two genotypes *_R_NKG2D rs1049174 GG* and *rs2255336 AA*. In patients exhibiting these two genotypes, immunosuppressive therapy based on mTOR inhibitors appears to be less effective in preventing ABMR compared to maintenance therapies based on CNIs [*rs1049174 GG*: 75.0% (15/20) mTOR vs. 35.7% (5/14) CNI; P = 0.035; OR: 0.2 (0.0 – 1.0) and *rs2255336 AA*: 66.6% (8/14) mTOR vs. 0% (0/4) CNI; P = 0.002; OR: 0.0 (0.0 – 0.3)]. Maintenance therapy does not appear to be influenced by the R/D 2 allele *MICA* mismatch (*2MM*), which nonetheless seems to have a synergistic effect in combination with the *rs1049174 GG* genotype (*2MM/GG+)*, further reducing the efficacy of mTOR inhibitor-based therapy [*2MM/GG+:* 100.0% (10/10) mTOR vs. 0% (0/2) CNI; P = 0.015; OR: 0.0 (0.0 – 0.7)]. The limited number of patients did not allow for analysis regarding the combination of R/D 2 allele *MICA* mismatch (*2MM*) with th*e rs2255336 AA* genotype *(2MM/AA+*).

**Table 6:** Correlation between maintenance therapy and the onset of antibody-mediated rejection (ABMR) based on the genetic profiles of kidney-transplanted patients.

Gene profiles of transplanted patients	Patients (N = 148)	Maintenance therapy	Total patients (N = 148)	SGF (N = 80)	ABMR (N = 68)	Comparisons of ABMR vs SGF		
						P value	OR	(95% CI)
*MICA alleles* 2MM	72	CsA/Tac ± MMF ± S	32	18	14	0.477	1.6	(0.6 – 4.4)
		Evl/Srl ± MMF ± S	40	18	22			
*rs1049174 GG*	34	CsA/Tac ± MMF ± S	14	9	5	**0.035**	0.2	(0.0 – 1.0)
		Evl/Srl ± MMF ± S	20	5	15			
*rs2255336 AA*	12	CsA/Tac ± MMF ± S	4	4	0	**0.002**	0.0	(0.0 – 0.3)
		Evl/Srl ± MMF ± S	12	0	8			
*MICA* 2MM + *rs1049174 GG*	12	CsA/Tac ± MMF ± S	2	2	0	**0.015**	0.0	(0.0 – 0.7)
		Evl/Srl ± MMF ± S	10	0	10			
*MICA* 2MM + *rs2255336 AA*	2	CsA/Tac ± MMF ± S	0	0	0	Not available		
		Evl/Srl ± MMF ± S	2	0	2			

CNI, Calcineurin inhibitors; mTOR, mammalian target of rapamycin; S, corticosteroids; CsA, cyclosporine A; Tac, tacrolimus; MMF, mycophenolate mofetil; Evl, Everolimus; Srl, sirolimus.

Bold values denote statistical significance at the p < 0.05 level.

## 4 Discussion

Despite the remarkable advancements in clinical and pharmacological fields in recent years, achieving complete graft tolerance remains challenging in solid organ transplantation. Antibody-mediated rejection stands as the primary immunological barrier, progressively compromising renal function, thus heightening the risk of morbidity and mortality in transplant recipients (32).

Extensive evidence supports that a higher level of compatibility between *HLA* class I and II in the recipient and donor correlates with improved graft function and survival (33). Moreover, MHC class I chain-related molecules A (*MICA*) may also play a significant role in kidney transplant outcomes, as recently highlighted by Carapito et al. (15) Our study provides a comprehensive and in-depth analysis of the genetic profile of these molecules in transplant patients, exploring their correlation with one of their main receptors: *NKG2D*.

An interesting finding that emerges from the study is the substantial overlap in *MICA* allele frequencies in transplanted patients compared to the control population, except for the MICA*002:01 allele, whose frequency is lower in patients (16% vs 24%). In the Sardinian population, this allele is in strong linkage disequilibrium (LD) (D’ = 1) with HLA-B*58:01, and both are part of the extended haplotype HLA-A*02:05, B*58:01, C*07:01, DRB1*03:01, which has a protective effect against microbiological infections, particularly SARS-CoV-2 infection (34). Furthermore, studies conducted in the Taiwanese population have shown that MICA*002 and MICA*009 play a protective role against the development of psoriasis and rheumatoid arthritis (35, 36). Therefore, the absence of this allele may have implications for the onset and progression of specific types of kidney diseases with an immunological dysregulation background.

Patients experiencing antibody-mediated rejection showed a significantly lower frequency of the MICA*010:01 allele (10 times less) compared to those with SGF. This allele is characterized by a proline-to-arginine substitution at position 6 of the alpha-1 domain, resulting in an unstable protein fold and the absence of cell surface expression (36, 37).

It has been shown that this protein remains trapped intracellularly, preventing the expression of functional MICA, whether in soluble or membrane-bound form (36). One possible explanation for this frequency imbalance between ABMR and SGF is that, due to its lack of expression, the MICA 10 peptide is unable to interact with the NKG2D receptor, thereby limiting immune response activation. This lack of activation may have a protective effect, as observed in Han Chinese populations carrying MICA*010 alleles, who exhibited a lower incidence of systemic lupus erythematosus (SLE) (36).

However, the significance of this finding is limited by the fact that Sardinia is a genetic isolate, and its population exhibits very homogeneous genetic characteristics, which are markedly different from Caucasian and American populations in the allele frequencies of both *HLA* class *I* and *II*, as well as *MICA* and *MICB* (38).

In line with the compelling work by Carapito et al. (15), this study underscores the significance of R/D *MICA*-allele matching to both the occurrence of ABMR episodes and the stable graft function. Graft survival is significantly affected by the number of *MICA* mismatches, with more mismatches correlating with progressively worse outcomes (0 > 1 > 2 MM). Specifically, the highest cumulative incidence of ABMR (52.7%) was observed in patients with two R/D MICA mismatches (**Figure 2A**, Log-rank = 0.03). Additionally, graft function deteriorated more rapidly in the presence of two *MICA* mismatches. This was evidenced by a more pronounced decline in eGFR, which achieved statistical significance at a later stage (P = 0.04 at 72 months post-transplantation) (**Supplementary Figures S2**, **S3**).

These data are consistent with the findings in other studies, including multicenter cohorts of kidney transplants, indicating that *MICA* allelic mismatches are associated with reduced graft survival and increased rejection (15). MICA antigens are not usually expressed in normal cells; however, they are overexpressed in renal, pancreatic, and heart allografts that undergo acute or antibody-mediated rejection (39, 40). The expression of MICA antigens may promote the development of “*de novo*” anti-MICA DSA, contributing to the onset of antibody-mediated rejection episodes and the progressive loss of graft function. Most likely these antibodies activate NK cells through the interaction with the CD16 receptor (15).

However, the implications of *MICA* mismatch in solid organ transplantation require further investigation. This necessity arises partly from the high polymorphism of *MICA* alleles, which can encode both membrane-bound forms and soluble isoforms. Consequently, different *MICA* alleles can exert varying biological functions by modulating the cytotoxic activity of NK cells and specific subsets of T cells in divergent ways (41). These variations can significantly impact graft rejection.

For example, the MICA*008 allele is characterized by the release of MICA molecules in exosomes, which downregulates the NKG2D receptor in NK cells. In contrast, alleles such as MICA*009 and MICA*002 encode antigens that are released via proteolytic cleavage and act as potent activators of NK cells through the NKG2D receptor (35, 36). Additionally, MICA*010 is not present on the cell membrane, suggesting its direct role in organ rejection may be limited. Indeed, in our study, this allele was found to be 10 times more frequent in patients with SGF compared to those with ABMR.

The study of MICA molecule mechanisms is further complicated by the fact that *MICA* alleles can encode molecules with methionine at position 129 (instead of valine) in the α2 domain. These methionine-encoded molecules exhibit a stronger binding affinity for the NKG2D receptor, leading to increased NK cell alloreactivity (42, 43).

Indeed, in our study, the highest risk of rejection was observed in R/D pairs with 2 *MICA* mismatches, particularly when both mismatches were represented by MICA-129 Methionine alleles (R/D: VV/MM; Log Rank < 0.001), (**Figure 2B**).

In these cases, donor MICA-129 methionine homozygosity appears to lead to high NK cell alloreactivity in the recipient (39, 40), negatively influencing transplant outcomes by increasing the incidence of ABMR (**Supplementary Figures S1A, B**).

The detrimental effect of the MICA-129 methionine polymorphism on kidney transplantation is further supported by the analysis of anti-MICA DSAs, which were found in 25% of patients who developed ABMR. In fact, the highest anti-MICA DSA titers (expressed as MFI > 1000) were observed for MICA antigens with Methionine at position 129. Specifically, in this study, the antigens that appear to be the most immunogenic are MICA 18 and MICA 01. A similar finding has been reported in previous studies, in which over 80% of patients with acute heart allograft rejection presented anti-MICA antigen-specific antibodies (measured by cytotoxicity) against MICA 01, followed by MICA 04, 11, and 18 (27.3%) (39).

A key finding of this study is the identification of a strong correlation between the high-cytotoxic *_R_NKG2D* genotypes (*s1049174* [GG] and *rs2255336* [AA]) and the worst renal outcomes, including the increase of cumulative incidence of ABMR and detrimental post-transplant renal function (**Figures 3**, **4**). The effect of the *rs1049174* [GG] genotype on the incidence of ABMR appears more significant than that of the *rs2255336* [AA] genotype (X² = 13.44; Log-rank = 0.001 vs X² = 0.34; Log-rank = 0.84). The *rs2255336* [AA] genotype has a frequency of less than 8.1% in transplanted patients, which likely leads to an underestimation of its impact due to the limited number of patients carrying this polymorphism.

Moreover, the detrimental effect on graft function from both polymorphisms (*rs1049174* [GG] and *rs2255336* [AA]) is markedly evident in the eGFR (P_AUC_ = 0.002 and P_AUC_= 0.028, respectively) and serum creatinine (SCr) (P_AUC_ = 0.023 and P_AUC_= 0.030, respectively) curves. Both of these SNPs are associated with high levels of NK cell cytotoxic activity mediated by the NKG2D receptor (43). However, their mechanisms differ: the *rs1049174* polymorphism enhances NKG2D mRNA transcription, leading to increased NKG2D expression *in vitro* (44), while the *rs2255336* substitution increases the receptor’s affinity for the DAP10 adaptor molecule when binding to NKG2D ligands (44, 45).

The heightened activation of the NKG2D receptor leads to increased cytotoxic activity by NK cells and specific T cell subsets, including NKT cells, CD8+ TCR-αβ, and CD4+ TCR-γδ T cells. Consequently, it is highly plausible that these specific genotypes play a substantial role in shaping the outcome of kidney transplantation, as indicated by the findings in this study. In hematopoietic stem cell transplantation (HSCT) also, the NKG2D receptor plays a significant role in NK cell cytotoxicity, influencing the transplantation outcome by causing complications such as graft-versus-host disease (GvHD) and post-transplant infections, as well as contributing to the beneficial graft-versus-leukemia (GvL) effect (43, 46, 47).

From a clinical perspective, the most exciting aspect of the study is evident from the analysis of the NKG2D/MICA pathway, which allowed the identification of specific patient categories at a higher risk of antibody-mediated rejection and rapid deterioration of renal function (**Figure 5**; **Supplementary Figures S8**, **S9**).

Indeed, patients at the highest risk of ABMR were those with homozygosity for the *_R_NKG2D rs1049174 [GG]* variant, in combination with either 2 *MICA* mismatches (*2MM/GG+*), or 1 MICA mismatch (*1MM/GG+*), (cumulative incidence 91.6% and 62.5% respectively). Conversely, the combination of *MICA* matching and the absence of the *_R_NKG2D rs1049174* [GG] variant *(0MM/GG-)* characterized patients with the lowest risk of ABMR (0MM/GG-: cumulative incidence 11.1%) (**Figure 5**).

It is important to note that the significant cytotoxic effect generated by the 2MM/GG+ combination (two *MICA* R/D mismatches and homozygosity at *rs1049174G* variant*)* manifests independently of R/D *HLA II* class match, as evidenced by analyzing the subgroup of patients with *HLA-DRB1* and *HLADQB1* full match (*X* ^2^ = 13.59; Log-rank = 0.001; **Figure 6**).

Furthermore, the impact of the 2MM/GG+ combination on the kidney transplant outcome remains independent of other clinical and genetic variables associated with antibody-mediated rejection, as highlighted by the multivariate logistic regression analysis (**Supplementary Table S4**). The two genetic variants, represented by the presence of two *MICA* R/D mismatches and homozygosity at *rs1049174 [GG]* allele, exert such a pronounced synergistic effect on NK cell-mediated alloreactivity that it reaches high statistical significance despite the limited number of examined patients.

In addition, it should be considered that MICA mismatches do not all have the same effect on NK cell activity. The presence of Methionine at codon position 129 (MICA-129) creates mismatches with high affinity for the NKG2D receptor, significantly influencing the alloreactivity of NK cells, including some subsets of T cells. This effect becomes evident when analyzing R/D MICA-129 mismatch combinations with the two high cytotoxic potential NKG2D receptor genotypes (*rs1049174* [GG] and *rs2255336* [AA]) (**Supplementary Figures S1A, B**). In fact, graft survival is rapidly compromised when R/D presents 2 MICA-129 mismatches (R/D VV/MM, MM/VV) and the recipient is NKG2D GG+ and/or AA+ (Log-Rank <0.001). These results are highly significant, but they are limited by the small number of patients and need to be validated in larger cohorts.

Although it has been addressed only marginally, one of the most exciting aspects of the study is the high risk of ABMR observed in patients with the genetic profiles *rs1049174 [*GG] and *rs2255336 [*AA] of the *NKG2D* gene (**Table 6**) when treated with maintenance regimens based on mTOR inhibitors (rapamycin, Everolimus) compared to the use of calcineurin inhibitors (cyclosporine and tacrolimus).

Treatment with mTOR inhibitors appears to be less effective in controlling alloreactivity induced by *NKG2D* receptors when they are more expressed (*rs1049174* [GG] and *rs2255336 [*AA] genotypes) or have high affinity for their ligands (44, 45). This reduced efficacy in countering antibody-mediated rejection with maintenance therapy based on mTOR inhibitors is even more apparent in the patient subgroup 2MM/GG+ (P = 0.015, **Table 6**).

Rapamycin and its derivatives, such as Everolimus, are allosteric inhibitors of mTOR, representing one of the main pathways for the proliferation, differentiation, and activation of NK cells (48–50).

Therefore, in our study, we would have expected a more effective immunosuppressive effect from maintenance therapy based on mTOR inhibitors. However, the reduced efficacy of mTOR inhibitors found in the study can be explained by the fact that NKG2D binding with its MICA ligand activates NK cells “via” the DAP10 signaling molecule. This cascade involves several molecular pathways (51), most independent of the mTOR signaling pathways mediated by phosphatidylinositol 3-hydroxy kinase PI3K (51). This hypothesis is supported by *in vitro* studies indicating that mTOR inhibitors are less effective than CNIs, both in reducing the expression of the C-type lectin receptors (NKG2A and NKG2D) and in the production and secretion of INF-gamma and other pro-inflammatory cytokines (50).

This could result in a progressive and continuous immunological insult to the graft caused by the activation of NK cells and some subsets of T cells such as NKT cells, CD8+ TCR-αβ, and CD4+ TCR-γδ T cells (52). In conclusion, the NKG2D/MICA combination appears to influence the outcome of kidney transplantation strongly. The study of the two polymorphisms, *rs1049174* and *rs2255336*, of the *NKG2D* gene and the molecular typing of *MICA* associated with screening for anti-MICA antibodies should be included in pre-transplant assessments. In patients with the *NKG2D rs1049174 [GG]* genotype at high risk of antibody-mediated rejection, special attention is necessary, and, where possible, efforts should be made to avoid transplantation with donors mismatched for both *MICA* alleles. Additionally, mTOR therapy seems less effective in limiting rejection onset in patients with this specific genetic profile (2MM/GG+). This observation could open the possibility of tailoring the immunosuppressive therapy scheme to prolong graft survival in the long term. The potential clinical and therapeutic implications are significant, underscoring the importance of confirming these results through multicenter studies conducted on larger and genetically diverse patient cohorts, especially considering the Sardinian population’s limited genetic polymorphism as a genetic isolate.

## Data Availability

The datasets presented in this study can be found in online repositories. The names of the repository/repositories and accession number(s) can be found below: PRJNA1077892(SRA).

## References

[1] Wolfe RA Ashby VB Milford EL Ojo AO Ettenger RE Agodoa LY. Comparison of mortality in all patients on dialysis, patients on dialysis awaiting transplantation, and recipients of a first cadaveric transplant. N Engl J Med. (1999) 341:1725–30. doi: 10.1056/NEJM199912023412303 10580071

[2] Merion RM Ashby VB Wolfe RA Distant DA Hulbert-Shearon TE Metzger RA. Deceased-donor characteristics and the survival benefit of kidney transplantation. JAMA. (2005) 294:2726–33. doi: 10.1001/jama.294.21.2726 16333008

[3] Kostro JZ Hellmann A Kobiela J Skóra I Lichodziejewska-Niemierko M Dębska-Ślizień A. Quality of life after kidney transplantation: A prospective study. Transplant Proc. (2016) 48:50–4. doi: 10.1016/j.transproceed.2015.10.058 26915842

[4] Tucker EL Smith AR Daskin MS Schapiro H Cottrell SM Gendron ES. Life and expectations post-kidney transplant: a qualitative analysis of patient responses. BMC Nephrol. (2019) 20:175. doi: 10.1186/s12882-019-1368-0 31096942 PMC6524208

[5] McCaughan JA Patterson CC Maxwell AP Courtney AE. Factors influencing survival after kidney transplant failure. Transplant Res. (2014) 3:18. doi: 10.1186/2047-1440-3-18 25276347 PMC4178314

[6] Boubaker K Bouabid B Bardi R Abderrahim E Ben Abdallah T Ayed KH. Immunological factors and renal allograft survival for more than fifteen years: a single center study from Tunisia. Saudi J Kidney Dis Transplant. (2006) 17:70–6. 17297543

[7] Poggio ED Augustine JJ Arrigain S Brennan DC Schold JD. Long-term kidney transplant graft survival-Making progress when most needed. Am J Transplant. (2021) 21:2824–32. doi: 10.1111/ajt.16463 33346917

[8] Lamb KE Lodhi S Meier-Kriesche HU. Long-term renal allograft survival in the United States: a critical reappraisal. Am J Transplant. (2011) 11:450–62. doi: 10.1111/j.1600-6143.2010.03283.x 20973913

[9] Tamargo CL Kant S. Pathophysiology of rejection in kidney transplantation. J Clin Med. (2023) 12:4130. doi: 10.3390/jcm12124130 37373823 PMC10299312

[10] Cornell LD. Histopathologic features of antibody mediated rejection: the Banff classification and beyond. Front Immunol. (2021) 12:718122. doi: 10.3389/fimmu.2021.718122 34646262 PMC8503253

[11] Bentall A Herrera LP Cornell LD Gonzales MA Dean PG Park WD. Differences in chronic intragraft inflammation between positive crossmatch and ABO-incompatible kidney transplantation. Transplantation. (2014) 98:1089–96. doi: 10.1097/TP.0000000000000188 24911035

[12] Haas M Rahman MH Racusen LC Kraus ES Bagnasco SM Segev DL. C4d and C3d staining in biopsies of ABO- and HLA-incompatible renal allografts: correlation with histologic findings. Am J Transplant. (2006) 6:1829–40. doi: 10.1111/j.1600-6143.2006.01356.x 16889542

[13] Littera R Piredda G Argiolas D Lai S Congeddu E Ragatzu P. KIR and their HLA Class I ligands: Two more pieces towards completing the puzzle of antibody-mediated rejection and graft loss in kidney transplantation. PloS One. (2017) 12:e0180831. doi: 10.1371/journal.pone.0180831 28686681 PMC5501603

[14] Choy MK Phipps ME. MICA polymorphism: biology and importance in immunity and disease. Trends Mol Med. (2010) 16:97–106. doi: 10.1016/j.molmed.2010.01.002 20153697

[15] Risti M Bicalho MD. MICA and NKG2D: is there an impact on kidney transplant outcome. Front Immunol. (2017) 8:179. doi: 10.3389/fimmu.2017.00179 28289413 PMC5326783

[16] Nowak I Magott-Procelewska M Kowal A Miazga M Wagner M Niepiekło-Miniewska W. Killer immunoglobulin-like receptor (KIR) and HLA genotypes affect the outcome of allogeneic kidney transplantation. PloS One. (2012) 7:e44718. doi: 10.1371/journal.pone.0044718 23028591 PMC3441441

[17] Carapito R Aouadi I Verniquet M Untrau M Pichot A Beaudrey T. The MHC class I MICA gene is a histocompatibility antigen in kidney transplantation. Nat Med. (2022) 28:989–98. doi: 10.1038/s41591-022-01725-2 PMC9117142 35288692

[18] Luo L Li Z Wu W Luo G Xu C Sun Z. Role of MICA antibodies in solid organ transplantation. Clin Transplant. (2014) 28:152–60. doi: 10.1111/ctr.2014.28.issue-2 24372774

[19] Luo L Li Z Wu W Luo G Mei H Sun Z. The effect of MICA antigens on kidney transplantation outcomes. Immunol Lett. (2013) 156:54–8. doi: 10.1016/j.imlet.2013.08.009 24004718

[20] Suárez-Alvarez B López-Vázquez A Baltar JM Baltar JM Ortega F López-Larrea C. Potential role of NKG2D and its ligands in organ transplantation: new target for immunointervention. Am J Transplant. (2009) 9:251–7. doi: 10.1111/j.1600-6143.2008.02526.x 19178412

[21] Hayashi T Imai K Morishita Y Hayashi I Kusunoki Y Nasaci K. Identification of the NKG2D haplotypes associated with natural cytotoxic activity of peripheral blood lymphocytes and cancer immunosurveillance. Cancer Res. (2006) 66:563–70. doi: 10.1158/0008-5472.CAN-05-2776 16397273

[22] Loupy A Haas M Roufosse C Naesens M Adam B Afrouzian M. The Banff 2019 Kidney Meeting Report (I): Updates on and clarification of criteria for T cell- and antibody-mediated rejection. Am J Transplant. (2020) 20:2318–31. doi: 10.1111/ajt.15898 PMC7496245 32463180

[23] Chowdhry M Makroo RN Singh M Kumar M Thakur Y Sharma V. Role of anti-MICA antibodies in graft survival of renal transplant recipients of India. J Immunol Res. (2018) 2018:3434050. doi: 10.1155/2018/3434050 29850626 PMC5907422

[24] Untergasser A Cutcutache I Koressaar T Ye J Faircloth BC Remm M. Primer3–new capabilities and interfaces. Nucleic Acids Res. (2012) 40:e115. doi: 10.1093/nar/gks596 22730293 PMC3424584

[25] Furue H Kumimoto H Matsuo K Suzuki T Hasegawa Y Shinoda M. Opposite impact of NKG2D genotype by lifestyle exposure to risk of aerodigestive tract cancer among Japanese. Int J Cancer. (2008) 123:181–6. doi: 10.1002/ijc.v123:1 18398831

[26] Vazquez-Gonzalez WG Martinez-Alvarez JC Arrazola-Garcia A Perez-Rodriguez M. Haplotype block 1 variant (HB-1v) of the NKG2 family of receptors. Hum Immunol. (2019) 80:842–7. doi: 10.1016/j.humimm.2019.07.276 31320124

[27] R core Team. R: A language and environment for statistical computing. Vienna, Austria: R Foundation for Statistical Computing (2023). Available at: https://www.R-project.org/.

[28] Barrett JC Fry B Maller J Daly MJ. Haploview: analysis and visualization of LD and haplotype maps. Bioinformatics. (2005) 21:263–5. doi: 10.1093/bioinformatics/bth457 15297300

[29] Lewontin RC. The interaction of selection and linkage. I. General considerations; Heterotic models. Genetics. (1964) 49:49–67. doi: 10.1093/genetics/49.1.49 17248194 PMC1210557

[30] Bland M ed. An Introduction to Medical Statistics. 4th ed. Oxford: Oxford University Press; Inc (2015).

[31] Jun KW Kim MH Hwang JK Kim SD Park SC Won YS. Impact of pretransplant panel-reactive antibody level on renal graft survival in patients with a negative crossmatch and no donor-specific antibody. Transplant Proc. (2016) 48:770–2. doi: 10.1016/j.transproceed.2015.12.099 27234732

[32] Boesmueller C Biebl M Scheidl S Oellinger R Margreiter C Pratschke J. Long-term outcome in kidney transplant recipients over 70 years in the Eurotransplant Senior Kidney Transplant Program: a single center experience. Transplantation. (2011) 92:210–6. doi: 10.1097/TP.0b013e318222ca2f 21642907

[33] Mohammadhassanzadeh H Oualkacha K Zhang W Klement W Bourdiec A Lamsatfi J. On path to informing hierarchy of eplet mismatches as determinants of kidney transplant loss. Kidney Int Rep. (2021) 6:1567–79. doi: 10.1016/j.ekir.2021.03.877 PMC8207474 34169197

[34] Littera R Campagna M Deidda S Angioni G Cipri S Melis M. Human leukocyte antigen complex and other immunogenetic and clinical factors influence susceptibility or protection to SARS-CoV-2 infection and severity of the disease course. Sardinian Experience Front Immunol. (2020) 11:605688. doi: 10.3389/fimmu.2020.605688 33343579 PMC7746644

[35] Ashiru O Boutet P Fernández-Messina L Agüera-González S Skepper JN Valés-Gómez M. Natural killer cell cytotoxicity is suppressed by exposure to the human NKG2D ligand MICA*008 that is shed by tumor cells in exosomes. Cancer Res. (2010) 70:481–9. doi: 10.1158/0008-5472.CAN-09-1688 PMC2817492 20068167

[36] Wang CM Tan KP Wu YJ Zheng JW Wu J Chen JY. Functional MICA variants are differentially associated with immune-mediated inflammatory diseases. Int J Mol Sci. (2024) 25:3036. doi: 10.3390/ijms25053036 38474281 PMC10931785

[37] Li Z Groh V Strong RK Spies T. A single amino acid substitution causes loss of expression of a MICA allele. Immunogenetics. (2000) 51:246–8. doi: 10.1007/s002510050039 10752636

[38] Birtsas V Batrinou A Dinou A Routsias J Gennimata V Iniotaki A. Distribution of MICA alleles and haplotypes associated with HLA-B in Greek population. Hum Immunol. (2021) 82:588–92. doi: 10.1016/j.humimm.2021.04.006 33966912

[39] Suárez-Alvarez B López-Vázquez A Gonzalez MZ Fdez-Morera JL Díaz-Molina B Blanco-Gelaz MA. The relationship of anti-MICA antibodies and MICA expression with heart allograft rejection. Am J Transplant. (2007) 7:1842–8. doi: 10.1111/j.1600-6143.2007.01838.x 17511763

[40] Hankey KG Drachenberg CB Papadimitriou JC Klassen DK Philosophe B Bartlett ST. MIC expression in renal and pancreatic allografts. Transplantation. (2002) 73:304–6. doi: 10.1097/00007890-200201270-00029 11821751

[41] Baranwal AK Mehra NK. Major histocompatibility complex class I chain-related A (MICA) molecules: relevance in solid organ transplantation. Front Immunol. (2017) 8:182. doi: 10.3389/fimmu.2017.00182 28293239 PMC5329007

[42] Steinle A Li P Morris DL Groh V Lanier LL Strong RK. Interactions of human NKG2D with its ligands MICA, MICB, and homologs of the mouse RAE-1 protein family. Immunogenetics. (2001) 53:279–87. doi: 10.1007/s002510100325 11491531

[43] Fuerst D Neuchel C Niederwieser D Bunjes D Gramatzki M Wagner E. Matching for the MICA-129 polymorphism is beneficial in unrelated hematopoietic stem cell transplantation. Blood. (2016) 128:3169–76. doi: 10.1182/blood-2016-05-716357 27811019

[44] Espinoza JL Nguyen VH Ichimura H Pham TT Nguyen CH Pham TV. A functional polymorphism in the NKG2D gene modulates NK-cell cytotoxicity and is associated with susceptibility to Human Papilloma Virus-related cancers. Sci Rep. (2016) 6:39231. doi: 10.1038/srep39231 27995954 PMC5172372

[45] Mariaselvam CM Tamouza R Krishnamoorthy R Charron D Misra DP Jain VK. Association of NKG2D gene variants with susceptibility and severity of rheumatoid arthritis. Clin Exp Immunol. (2017) 187:369–75. doi: 10.1111/cei.12891 PMC5290229 27783394

[46] Marçais A Cherfils-Vicini J Viant C Degouve S Viel S Fenis A. The metabolic checkpoint kinase mTOR is essential for IL-15 signaling during the development and activation of NK cells. Nat Immunol. (2014) 15:749–57. doi: 10.1038/ni.2936 PMC4110708 24973821

[47] Pontrelli P Rascio F Castellano G Grandaliano G Gesualdo L Stallone G. The role of natural killer cells in the immune response in kidney transplantation. Front Immunol. (2020) 11:1454. doi: 10.3389/fimmu.2020.01454 32793200 PMC7390843

[48] Liu Q Thoreen C Wang J Sabatini D Gray NS. mTOR mediated anti-cancer drug discovery. Drug Discovery Today Ther Strateg. (2009) 6:47–55. doi: 10.1016/j.ddstr.2009.12.001 PMC2901551 20622997

[49] Siemaszko J Marzec-Przyszlak A Bogunia-Kubik K. NKG2D natural killer cell receptor-A short description and potential clinical applications. Cells. (2021) 10:1420. doi: 10.3390/cells10061420 34200375 PMC8229527

[50] Mao B Zhang Q Ma L Zhao DS Zhao P Yan P. Overview of Research into mTOR Inhibitors. Molecules. (2022) 27:5295. doi: 10.3390/molecules27165295 36014530 PMC9413691

[51] Pradier A Papaserafeim M Li N Rietveld A Kaestel C Gruaz L. Small-molecule immunosuppressive drugs and therapeutic immunoglobulins differentially inhibit NK cell effector functions *in vitro* . Front Immunol. (2019) 10:556. doi: 10.3389/fimmu.2019.00556 30972058 PMC6445861

[52] Almeida JS Casanova JM Santos-Rosa M Tarazona R Solana R Rodrigues-Santos P. Natural killer T-like cells: immunobiology and role in disease. Int J Mol Sci. (2023) 24:2743. doi: 10.3390/ijms24032743 36769064 PMC9917533

