# Supplementary material for: MICA and NKG2D gene polymorphisms influence graft survival, and response to therapy in kidney transplantation
Source: Front Immunol. 2024 Nov 7;15:1440887. doi: 10.3389/fimmu.2024.1440887 (PMC11578996; doi:10.3389/fimmu.2024.1440887)
Supplement: Supplementary file 1 [file DataSheet1.pdf]

Supporting Information

Supplementary figures legends.

Figure S1A: Cumulative incidence of antibody-mediated rejection in patients according to the MICA-129 polymorphism and *$_R$NKG2D [GG] (rs1049174).*

The cumulative incidence of rejection events is graphically presented for a cohort of 52 R/D pairs based on the *MICA-129* genotype mismatch and ***$_R$NKG2D [GG] (rs1049174)*** observed over 120 months.

Patients were categorized into three groups based on the following criteria:

1. *R/D MM/VV / RNKG2D GG+ (red)*
2. *R/D VV/MM / RNKG2D GG+ (purple)*
3. *R/D VV/MM / RNKG2D GG- (green)*
4. *R/D MM/VV / RNKG2D GG- (orange)*
5. *R/D MM/MV / RNKG2D GG- (grey)*
6. *R/D VV/MV / RNKG2D GG- (black)*
7. *R/D MM/MV / RNKG2D GG+ (light grey)*
8. *R/D VV/MV / RNKG2D GG+ (light grey)*

P-values were calculated using the two-sided Log-rank test without correction. $\chi 2$: Chi-square.

Figure S1B: Cumulative incidence of antibody-mediated rejection in patients according to the MICA-129 polymorphism and [*AA] (rs2255336).*

The cumulative incidence of rejection events is graphically presented for a cohort of 52 R/D pairs based on the *MICA-129* genotype mismatch and ***$_R$NKG2D AA] (rs2255336)*** observed over 120 months. Patients were categorized into three groups based on the following criteria:

1. *R/D MM/VV / RNKG2D AA+ (red)*
2. *R/D VV/MM / RNKG2D AA- (green)*
3. *R/D MM/VV / RNKG2D AA- (orange)*
4. *R/D MM/MV / RNKG2D AA- (grey)*
5. *R/D VV/MV / RNKG2D AA- (black)*
6. *R/D VV/MM / RNKG2D AA+ (light grey)*
7. *R/D MM/MV / RNKG2D AA+ (light grey)*
8. *R/D VV/MV / RNKG2D AA+ (light grey)*

P-values were calculated using the two-sided Log-rank test without correction. $\chi 2$: Chi-square.

Figure S2: Estimated Glomerular Filtration Rate (eGFR) at 1, 6, 12, 36, and 72 Months After Transplantation in KTPs according to Recipient-Donor (R/D**) *MICA* allele mismatches.** The error bar at each point represents the relative error. The group of patients was stratified into three groups of patients according to donor-recipient *MICA* allele mismatches [0MM (black), 1MM (green), 2 MM (red)]. P-values were obtained by comparing groups of stratified patients by computing the area under the curve (AUC) for the respective plots. MM: Mismatches. eGFR ml/min/1.73m2. Statistically significant differences between 2MM, 1MM and 0 MM at 36 months [(57.9 ± 25.62 mL/min/1.73 m^2 (2 MM) vs 68.56 ± 25.76 mL/min/1.73 m^2 (1 MM) vs 74.35 ± 28.99 mL/min/1.73 m^2 (0 MM); P = 0.11]; at 72 months [(54.28 ± 28.92 mL/min/1.73 m^2 (2 MM) vs 57.32 ± 33.1 mL/min/1.73 m^2 (1 MM) vs 75.6 ± 33.62 mL/min/1.73 m^2 0 MM); P = 0.04] transplantation , P_{AUC} = 0.106.

Figure S3: Serum creatinine levels (µmol/L) at 1, 6, 12, 36, and 72 Months After Transplantation in KTPs according to recipient donor (R/D**) *MICA* allele mismatches.** The error bar at each point represents the relative error. The group of patients was stratified into three groups of patients according to donor-recipient *MICA* allele mismatches [0MM (black), 1MM (green), 2 MM (red)]. P-values were obtained by comparing groups of stratified

patients by computing the area under the curve (AUC) for the respective plots. MM: Mismatches. Statistically significant differences between 2MM, 1MM and 0 MM at 12 months [(119.16 ± 66.78 µmol/L (2 MM) vs 112.91 ± 57.92 µmol/L (1 MM) vs 88.98 ± 18.41 µmol/L (0 MM); P = 0.14], 36 months [(140.78 ± 152.34 µmol/L (2 MM) vs 106.24 ± 50.07 µmol/L (1 MM) vs 103.15 ± 63.54 µmol/L (0 MM); P = 0.47], at 72 months [(191.01 ± 203.43 µmol/L (2 MM) vs 181.86 ± 212.84 µmol/L (1 MM) vs 94.13 ± 57.79 µmol/L (0 MM); P = 0.12] after transplantation, P_{AUC} = 0.042.

Figure S4: Estimated Glomerular filtration rate (eGFR) at 1, 6, 12, 36, and 72 months after transplantation in KTPs according to *NKG2D rs1049174 (G>C)* genotype. The error bar at each point represents the relative error. The group of patients was stratified into three groups of patients according to *NKG2D rs1049174 (G/C)* genotype [GG (red), GC (green), CC (black)]; P-values were obtained by comparing groups of stratified patients through the computation of the area under the curve (AUC) for the respective plots. eGFR ml/min/1.73m2.
Statistically significant differences between *rs1049174* [GG], [CG]and [CC] genotypes at 12 months [(57.90 ± 25.98 mL/min/1.73 m^2 [GG] vs 63.34 ± 22.74 mL/min/1.73 m^2 [CG] vs 71.22 ± 21.93 mL/min/1.73 m^2 [CC]; P = 0.085], at 36 months [(52.42 ± 23.22 mL/min/1.73 m^2 [GG] vs 58.23 ± 26.92 mL/min/1.73 m^2 [CG] vs 77.60 ± 24.74 mL/min/1.73 m^2 [CC]; P = 0.007], at 72 months [(42.60 ± 27.43 mL/min/1.73 m^2 [GG] vs 63.18 ± 30.48 mL/min/1.73 m^2[CG] vs 64.02 ± 31.50 mL/min/1.73 m^2 [CC]; P = 0.037] after transplantation, P_{AUC} = 0.002.

Figure S5: Serum creatinine levels (µmol/L) after transplantation at 1, 6, 12, 36, and 72 months after transplantation in KTPs according to *NKG2D rs1049174 (G>C)* genotype. The error bar at each point represents the relative error. The group of patients was stratified into three groups of patients according to *NKG2D rs1049174 (G/C)* genotype [GG (red), GC (green), CC (black)]; P-values were obtained by comparing groups of stratified patients through the computation of the area under the curve (AUC) for the respective plots. Statistically significant differences between r*s1049174* [GG], [CG]and [CC] genotypes at 12 months [(128.65 ± 64.52 µmol/L [GG] vs 121.20 ± 73.37 µmol/L [CG] vs 99.28 ± 28.67 µmol/L [CC]; P = 0.046], at 36 months [(140.14 ± 65.93 µmol/L [GG] vs 122.52 ± 57.24 µmol/L [CG] vs 92.55 ± 35.97 µmol/L [CC]; P = 0.009], at 72 months [(195.58 ±121.70 µmol/L [GG] vs 137.80 ± 168.75 µmol/L [CC] vs 125.87 ± 89.65 µmol/L [CC]; P = 0.042], after transplantation, P_{AUC} = 0.023.

Figure S6: Estimated Glomerular filtration rate (eGFR) at 1, 6, 12, 36, and 72 months after transplantation in KTPs according to *NKG2D rs2255336 (A>G)* **genotype.** The error bar at each point represents the relative error. The patient cohort was stratified into three groups based on the presence of the *NKG2D rs2255336 (A>G)* [*AA (light blue), AG (green), GG (red)]*. P-values were obtained by comparing groups of stratified patients by computing the area under the curve (AUC) for the respective plots. eGFR ml/min/1.73m2. Statistically significant differences between *rs2255336* [AA], [AG] and [GG] genotypes at 12 months [(55.60 ± 32.13 mL/min/1.73 m^2 [AA] vs 63.40 ± 23.58 mL/min/1.73 m^2[AG]- vs 69.41 ± 21.31 mL/min/1.73 m^2 [GG]; P = 0.194] at 36 months [(39.31 ± 20.38 ml/min [AA] vs 60.45 ± 28.05 mL/min/1.73 m^2 (AG) vs 73.53 ± 24.29 mL/min/1.73 m^2 [GG]; P = 0.047] and at 72 months [(26.34 ± 20.32 mL/min/1.73 m^2 [GG] vs 62.10 ± 31.89 mL/min/1.73 m^2 [AG] vs 61.45 ± 29.28 mL/min/1.73 m^2 [GG]; P = 0.015] after transplantation P_{AUC} = 0.028).

Figure S7: Serum creatinine levels (µmol/L) after transplantation measured at 1, 6, 12, 36, and 72 months after transplantation in KTPs according to *NKG2D rs2255336 (A>G)* **genotype.** The error bar at each point represents the relative error. The patient cohort was stratified into three groups based on the presence of the *NKG2D rs2255336 (A>G)* [*AA (light blue), AG (green), GG (red)]*. P-values were obtained by comparing groups of stratified patients by computing the area under the curve (AUC) for the respective plots. Statistically significant differences between *rs2255336* [AA], [AG] and [GG] genotypes at 12 months [(154.13 ± 89.45 µmol/L [AA] vs 119.19 ± 67.38 µmol/L [AG] -vs 100.31 ± 27.70 µmol/L [GG]; P = 0.073], at 36 months [(189.38 ± 68.47 µmol/L [AA] vs 148.80 ± 154.93 µmol/L [AG] vs 95.95 ± 35.32 µmol/L [GG]; P = 0.307] and at 72 months [(286.55 ± 154.16 µmol/L [AA] vs 170.28 ± 216.80 µmol/L [AG] vs 152.21 ± 165.73 µmol/L [GG]; P = 0.160] after transplantation, P_{AUC} = 0.030.

Figure S8: Estimated Glomerular filtration rate (eGFR) at 1, 6, 12, 36, 72 months after transplantation in KTPs according to *NKG2D* rs1049174 [GG] genotype and *MICA* allele mismatches. The error bar at each point

represents the relative error. The patient cohort was stratified into six groups based on the presence or absence of the GG (*rs1049174)* genotype and the donor-recipient *MICA* allele mismatches: 1. Two *MICA* R/D allele mismatches and *rs1049174* $_R$*NKG2D[GG] (purple)* 2. One *MICA* R/D allele mismatches and *rs1049174* $_R$*NKG2D[GG] (green)* 3. One *MICA* R/D allele mismatches and *rs1049174* $_R$*NKG2D[GG] (orange)* 4. Two *MICA* R/D allele mismatch and *rs1049174* $_R$*NKG2D[CG]* and *[CC] (light blue)* 5. *MICA* R/D alleles match and *rs1049174* $_R$*NKG2D[GG] (red)* 6. *MICA* R/D alleles match and *rs1049174* $_R$*NKG2D[CG]* and *[CC] (black).*

P-values were obtained by comparing groups of stratified patients by computing the area under the curve (AUC) for the respective plots. MM: mismatches.

Statistically significant differences between 2MM/GG+, 1MM/GG+, lMM/GG-, 2MM/GG- and 0MM/GG+, 0MM/GG-: at 12 months [(56.60 ± 25.54 mL/min/1.73 m^2 vs 63.60 ± 23.15 mL/min/1.73 m^2 vs 83.14 ± 16.74 mL/min/1.73 m^2 respectively; P = 0.036], at 36 months [(51.71 ± 26.25mL/min/1.73 m^{2}vs 63.91 ± 26.95 mL/min/1.73 m^2 vs 84.48 ± 21.97 mL/min/1.73 m^2; P = 0.040] and at 72 months [(33.52 ± 26.45 mL/min/1.73 m^2 vs 57.52 ± 29.44 mL/min/1.73 m^2 vs 86.94 ± 25.56 mL/min/1.73 m^2; P = 0.002] after transplantation, P_{AUC} = 0.013).

Figure S9: Serum creatinine levels (µmol/L) after transplantation at 1, 6, 12, 36, 72 months after transplantation in KTPs according to *NKG2D* rs1049174 [GG] genotype and *MICA* allele mismatches. The error bar at each point represents the relative error. The patient cohort was stratified into six groups based on the presence or absence of the GG (*rs1049174)* genotype and the donor-recipient *MICA* allele mismatches: 1. Two *MICA* R/D allele mismatches and *rs1049174* $_R$*NKG2D[GG] (purple)* 2. One *MICA* R/D allele mismatches and *rs1049174* $_R$*NKG2D[GG] (green)* 3. One *MICA* R/D allele mismatches and *rs1049174* $_R$*NKG2D[GG] (orange)* 4. Two *MICA* R/D allele mismatch and *rs1049174* $_R$*NKG2D[CG]* and *[CC] (light blue)* 5. *MICA* R/D alleles match and *rs1049174* $_R$*NKG2D[GG] (red)* 6. *MICA* R/D alleles match and *rs1049174* $_R$*NKG2D[CG]* and *[CC] (black).*

P-values were obtained by comparing groups of stratified patients by computing the area under the curve (AUC) for the respective plots. MM: mismatches. Statistically significant differences between 2MM/GG+, 1MM/GG+, lMM/GG-, 2MM/GG- and 0MM/GG+, 0MM/GG at 12 months[(129.50 ± 48.98 µmol/L vs 117.43 ± 61.07 µmol/L vs 80.86 ± 14.27 µmol/L respectively; P = 0.019]; at 36 months [(142.83 ± 63.47 µmol/L vs 131.70 ± 125.65 µmol/L vs 81.67 ± 20.72 µmol/L respectively; P = 0.036] and at 72 months [(241.74 ± 155.35 µmol/L vs 178.94 ± 205.18 µmol/L vs 70.80 ± 18.32 µmol/L respectively; P = 0.008], P_{AUC} = 0.018.

Figure S10: Cumulative incidence of antibody-mediated rejection in patients *HLA-DRB1* and *HLA-DQB1* 1-4 mismatches R/D according to NKG2D genotype and MICA allele mismatches.

The cumulative incidence of rejection events is graphically presented for a cohort of 133 patients with *HLA-DRB1* and *HLA-DQB1* 1-4 mismatches R/D observed over 120 months.

Patients were categorized into four groups based on the following criteria:

1. R/D *MICA* alleles match independently of the *NKG2D rs1049174* genotype (*black).*

2. R/D *MICA* alleles 1-2 mismatches with *NKG2D rs1049174 CG* or *CC* genotype (marked as GG-) *(green).*

3. R/D *MICA* alleles 1 mismatch with *NKG2D rs1049174 GG* genotype *(light blue).*

4. R/D *MICA* alleles 2 mismatches with N*KG2D rs1049174 GG* genotype (*red).*

P-values were calculated using the two-sided Log-rank test without correction. $\chi 2$: Chi-square. MM: mismatches; R/D: recipient-donor.

Figure S11: Cumulative incidence of chronic rejection in patients HLA-A,-B,-C ≤3 mismatches according R/D to NKG2D genotype and MICA allele mismatches.

The cumulative incidence of rejection events is graphically presented for a cohort of 55 patients with ***HLA-A,-B,-C ≤3* mismatches** R/D observed over 120 months.

Patients were categorized into three groups based on the following criteria:

1. R/D *MICA* alleles match independently of the *NKG2D rs1049174* genotype (*black)*.

2. R/D *MICA* alleles 1-2 mismatches with *NKG2D rs1049174 CG* or *CC* genotype (marked as GG-) *(green)*.

3. R/D *MICA* alleles 1-2 mismatch with *NKG2D rs1049174 GG* genotype (*red)*.

P-values were calculated using the two-sided Log-rank test without correction. χ2: Chi-square. MM: mismatches; R/D: recipient-donor.

Figure S12: Cumulative incidence of antibody-mediated rejection in patients HLA-A,-B,-C 4-6 mismatches R/D according to NKG2D genotype and MICA allele mismatches.

The cumulative incidence of rejection events is graphically presented for a cohort of 93 patients with ***HLA-A,-B,-C 4-6*** **mismatches** R/D observed over 120 months.

Patients were categorized into three groups based on the following criteria:

1. R/D *MICA* alleles match independently of the *NKG2D rs1049174* genotype (*black)*.

2. R/D *MICA* alleles 1-2 mismatches with *NKG2D rs1049174 CG* or *CC* genotype (marked as GG-) *(green)*.

3. R/D *MICA* alleles 1-2 mismatch with *NKG2D rs1049174 GG* genotype (*red)*.

P-values were calculated using the two-sided Log-rank test without correction. χ2: Chi-square. MM: mismatches; R/D: recipient-donor.

Supplementary figures

Figure S1A

Kidney ABMR: *MICA-129* R/D Mismatches and $_R$*NKG2D [GG] (rs1049174)*

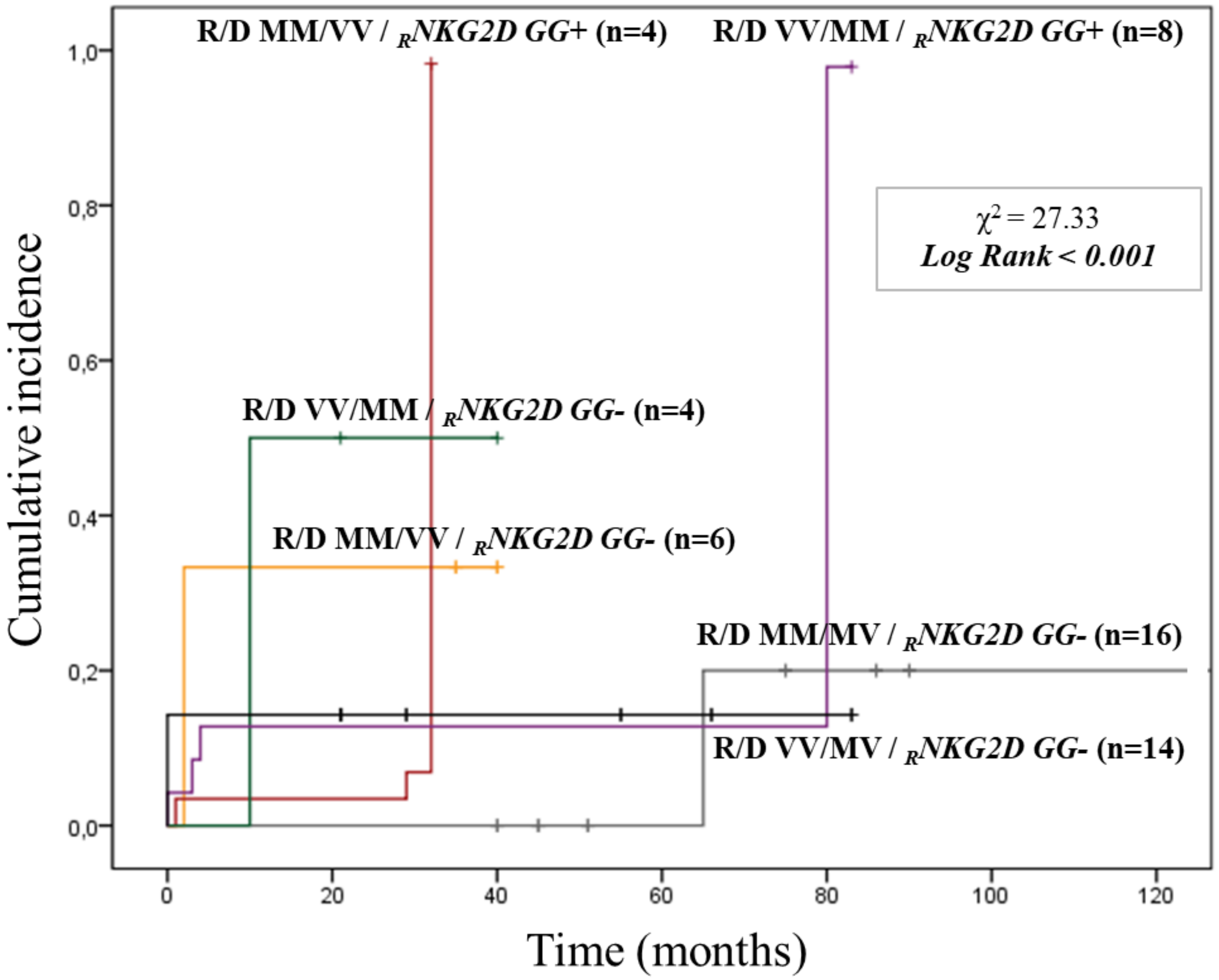

Time (Months)	0	20	40	60	80	100	120
R/D MM/VV / $_R$NKG2D GG+	4	2	0	0	0	0	0
R/D VV/MM / $_R$NKG2D GG+	8	2	2	2	0	0	0
R/D VV/MM / $_R$NKG2D GG-	4	2	0	0	0	0	0
R/D MM/VV / $_R$NKG2D GG-	6	4	3	1	0	0	0
R/D MM/MV / $_R$NKG2D GG-	16	16	15	12	8	4	2
R/D VV/MV / $_R$NKG2D GG-	14	12	6	4	2	0	0
R/D MM/MV / $_R$NKG2D GG+	0	-	-	-	-	-	-
R/D VV/MV / $_R$NKG2D GG+	0	-	-	-	-	-	-

Number at risk

Figure S1B

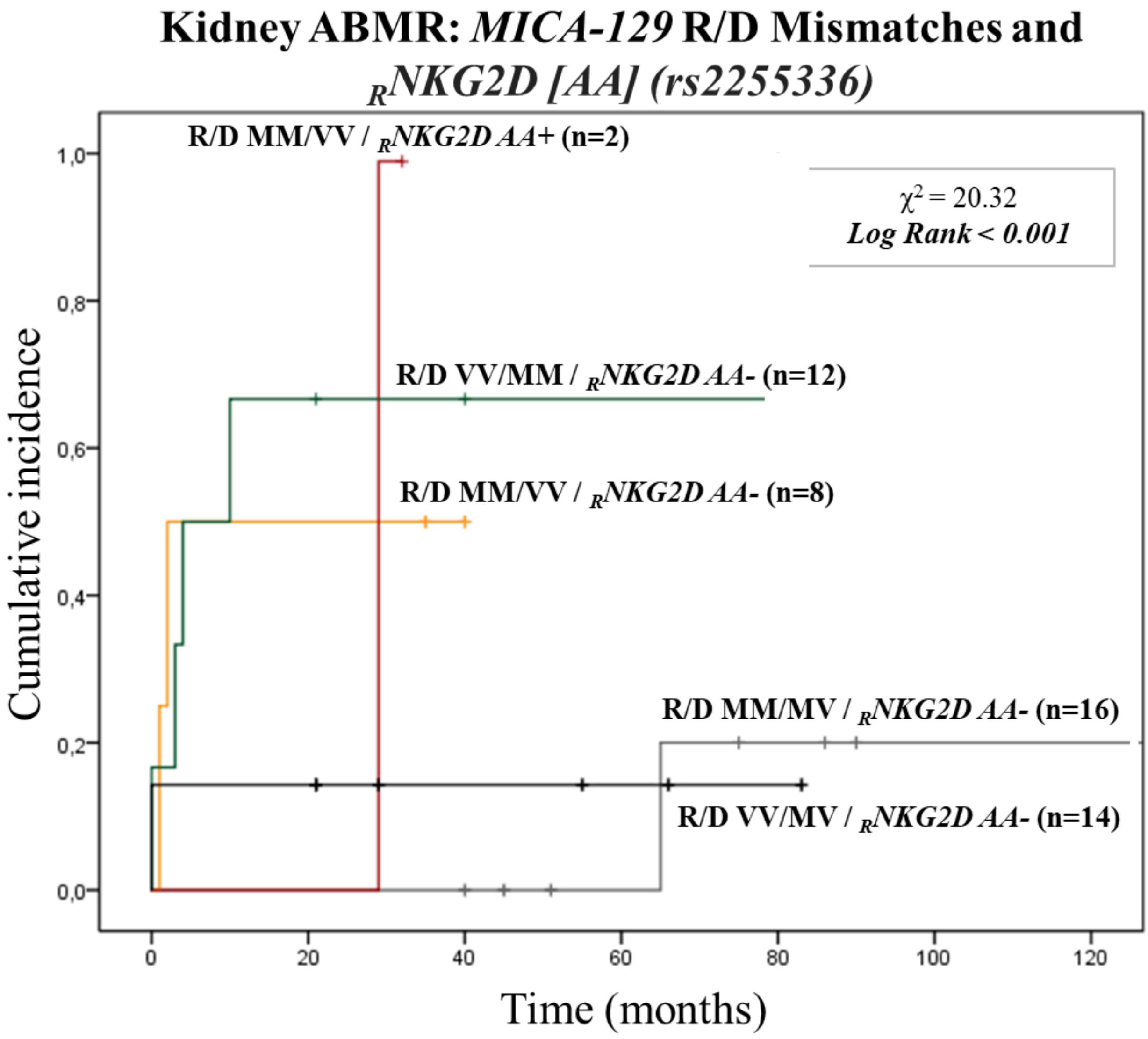

Time (Months)	0	20	40	60	80	100	120	
R/D MM/VV / $_R$NKG2D AA+	2	2	0	0	0	0	0	**Number at risk**
R/D VV/MM / $_R$NKG2D AA-	12	4	1	1	1	1	0	
R/D MM/VV / $_R$NKG2D AA-	8	4	1	0	0	0	0	
R/D MM/MV / $_R$NKG2D AA-	16	16	14	10	6	2	2	
R/D VV/MV / $_R$NKG2D AA-	14	12	6	4	2	0	0	
R/D VV/MM / RNKG2D AA+	0	-	-	-	-	-	-	
R/D MM/MV / $_R$NKG2D AA+	0	-	-	-	-	-	-	
R/D VV/MV / $_R$NKG2D AA+	0	-	-	-	-	-	-	

Figure S2

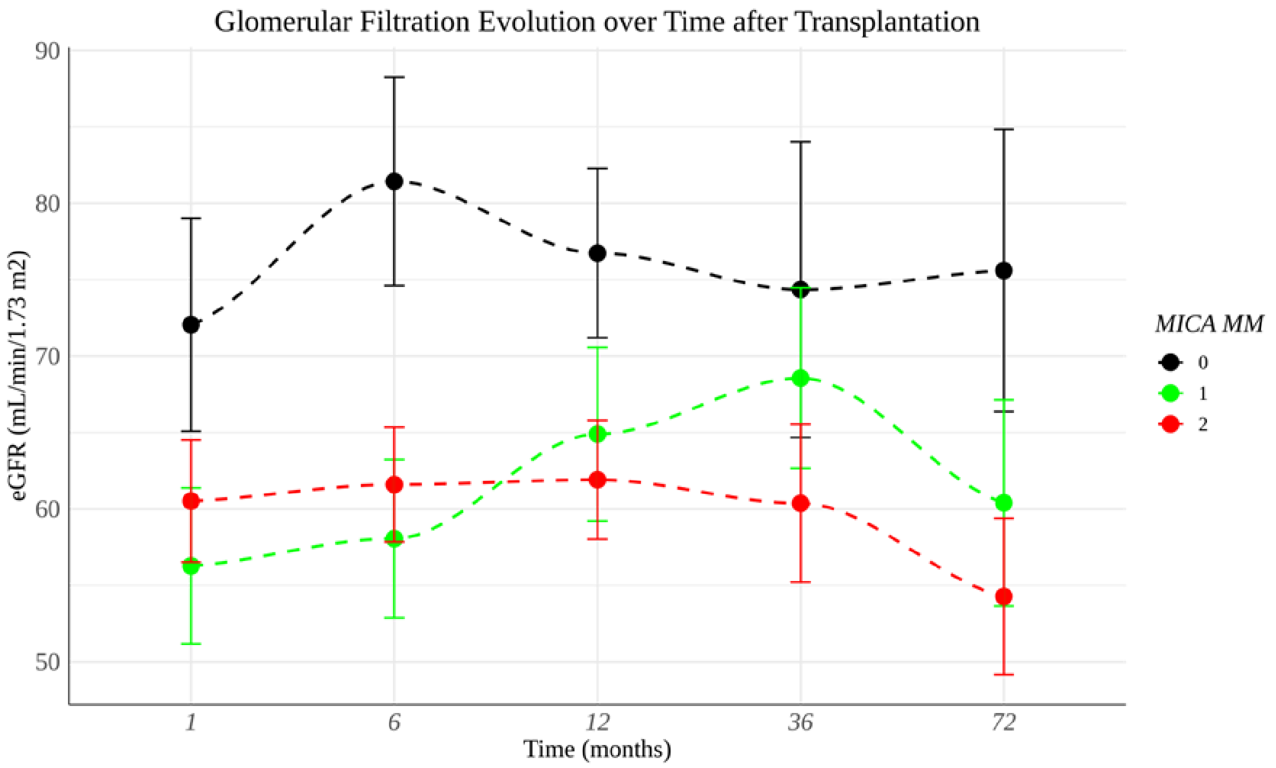

Figure S3

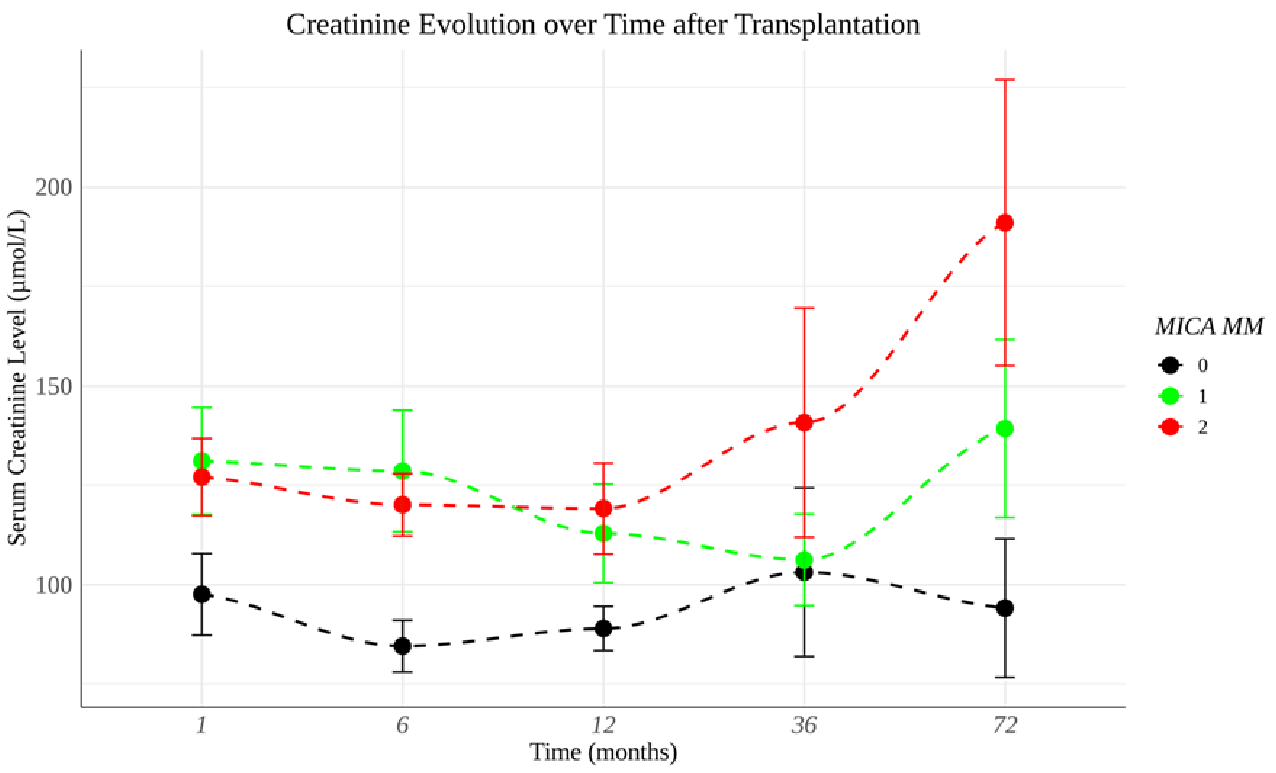

Figure S4.

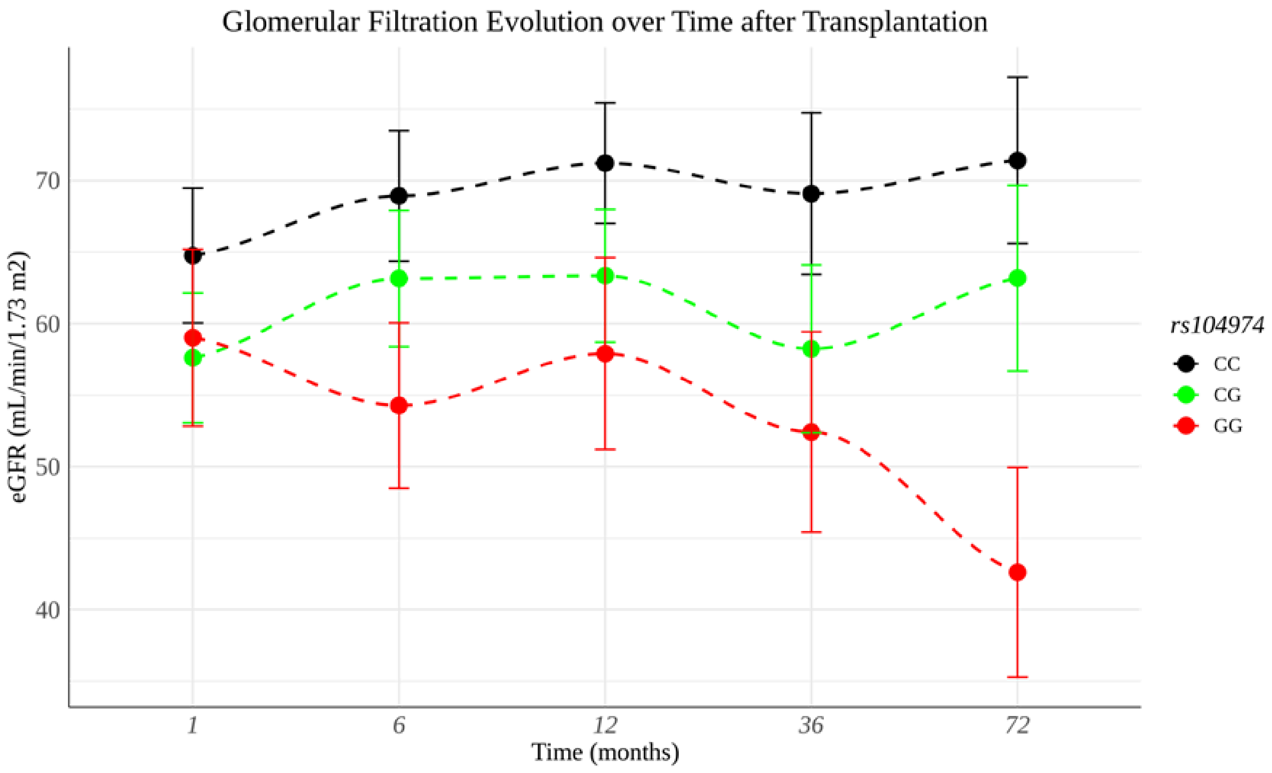

Figure S5.

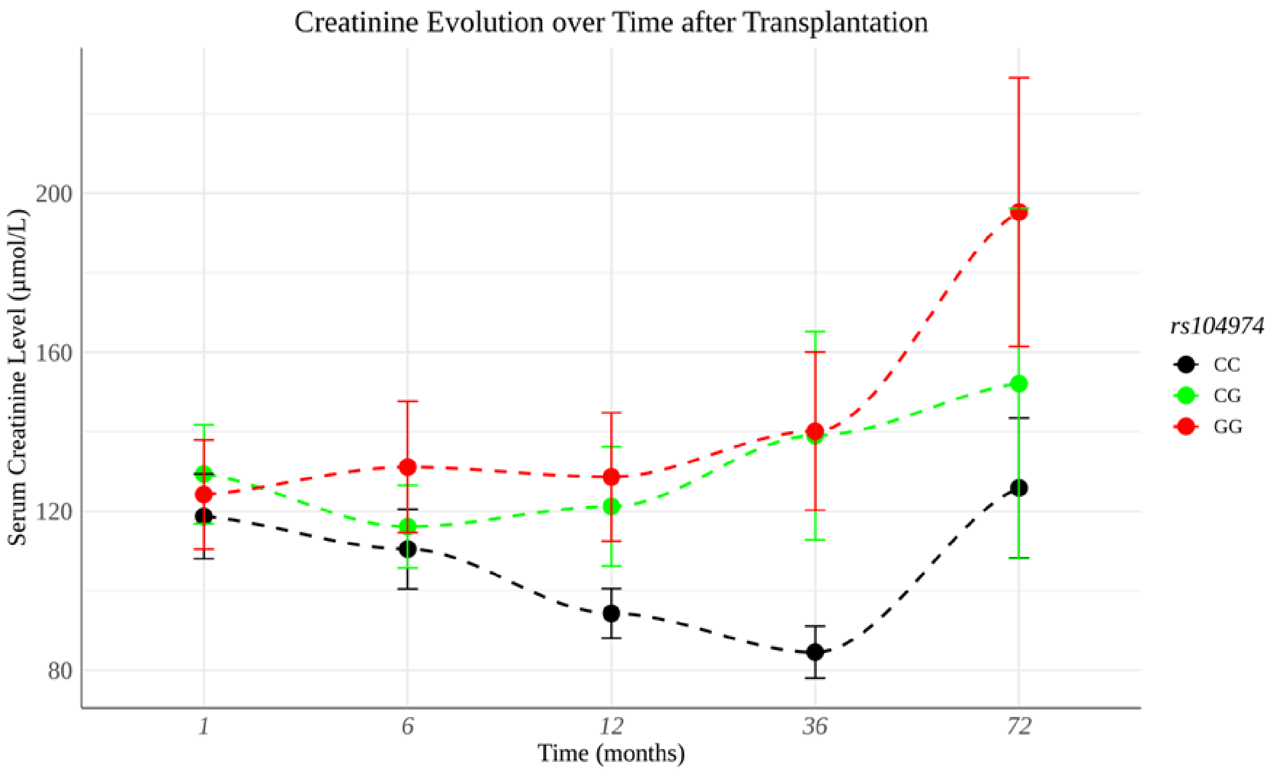

Figure S6.

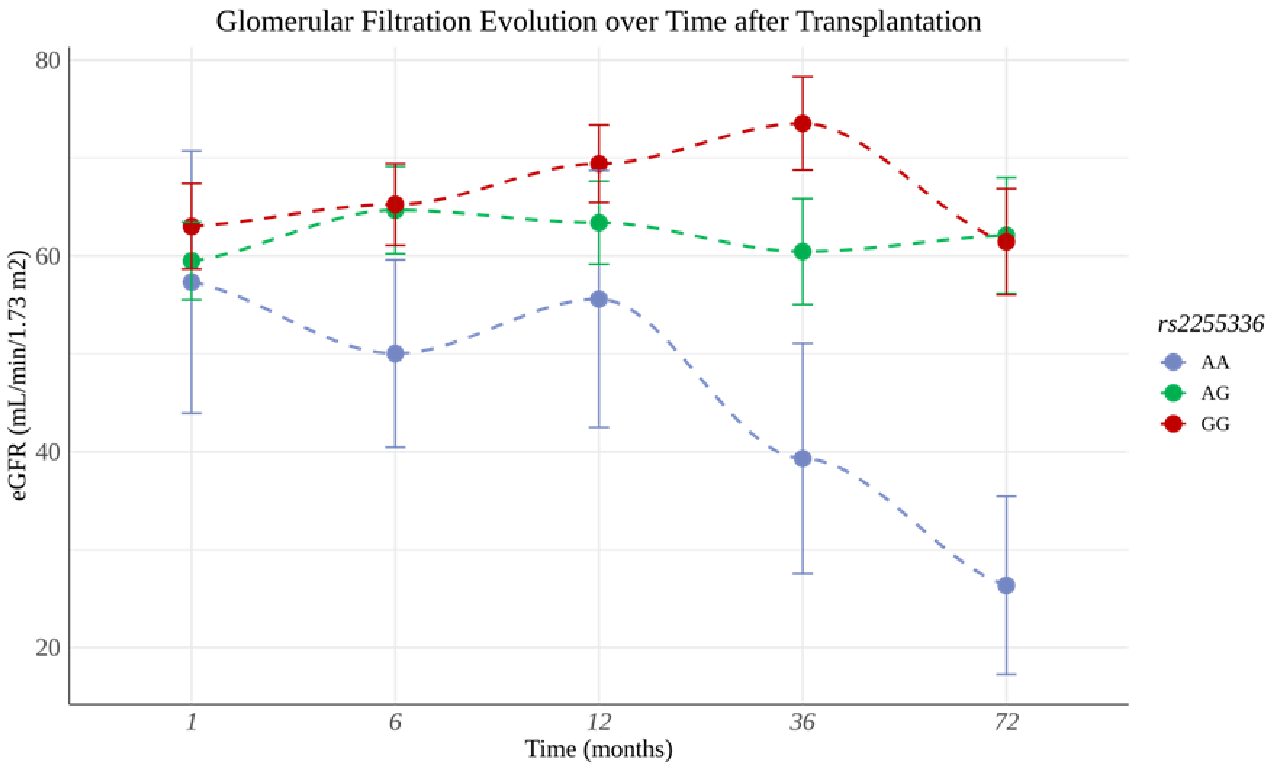

Figure S7.

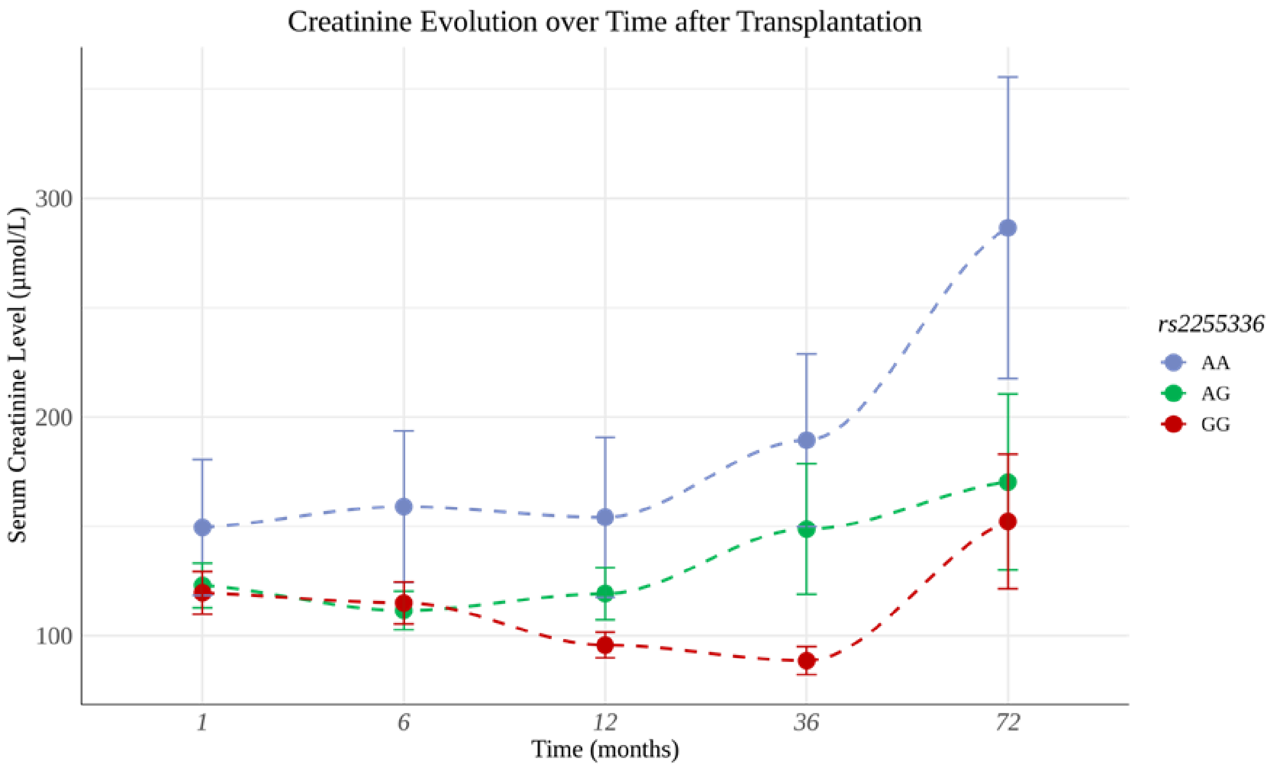

Figure S8

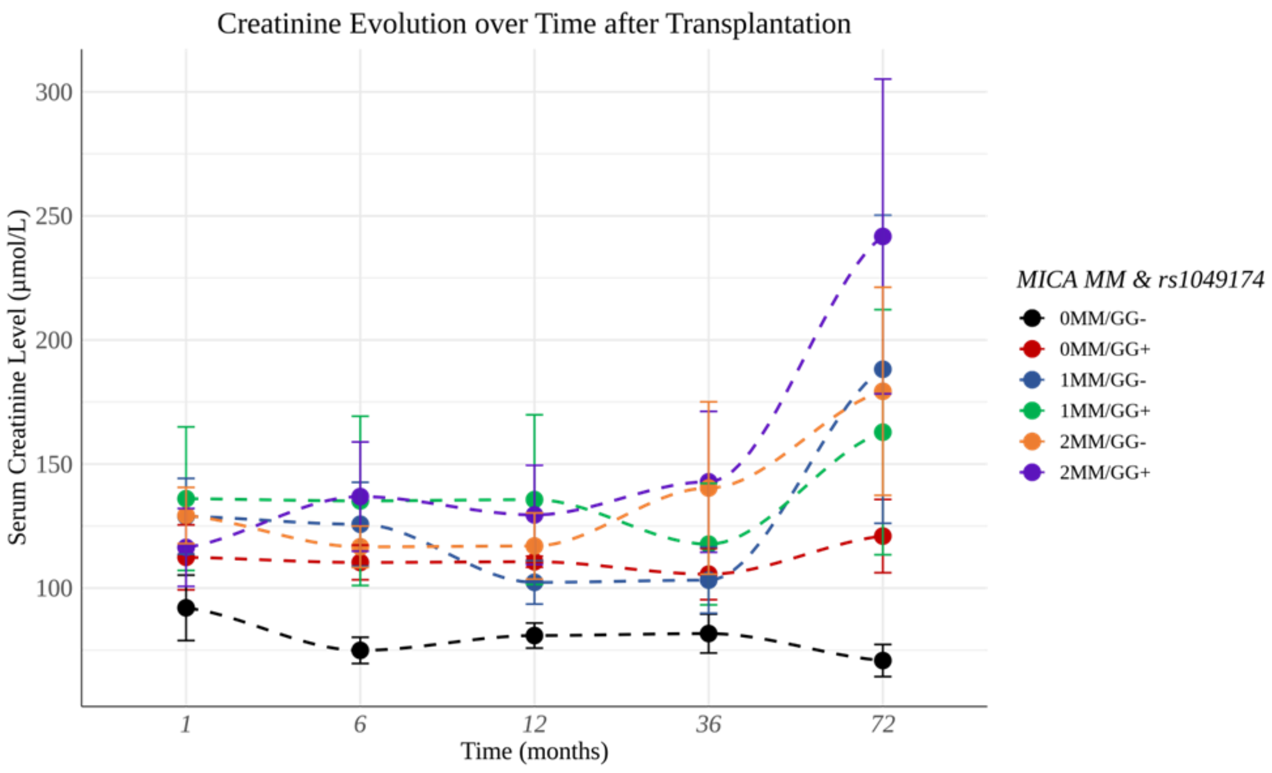

Figure S9.

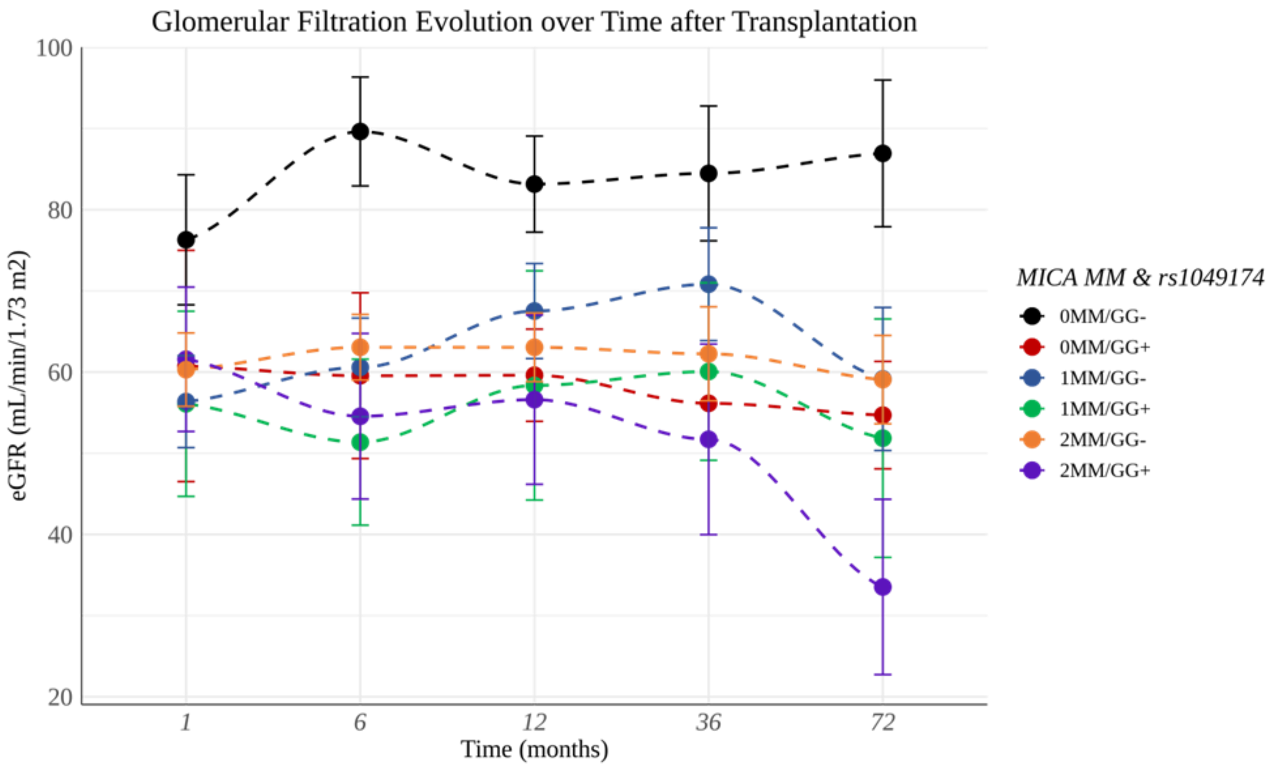

Figure S10

Kidney chronic rejection in *HLA-DRB1* and *HLA-DQB1* mismatch

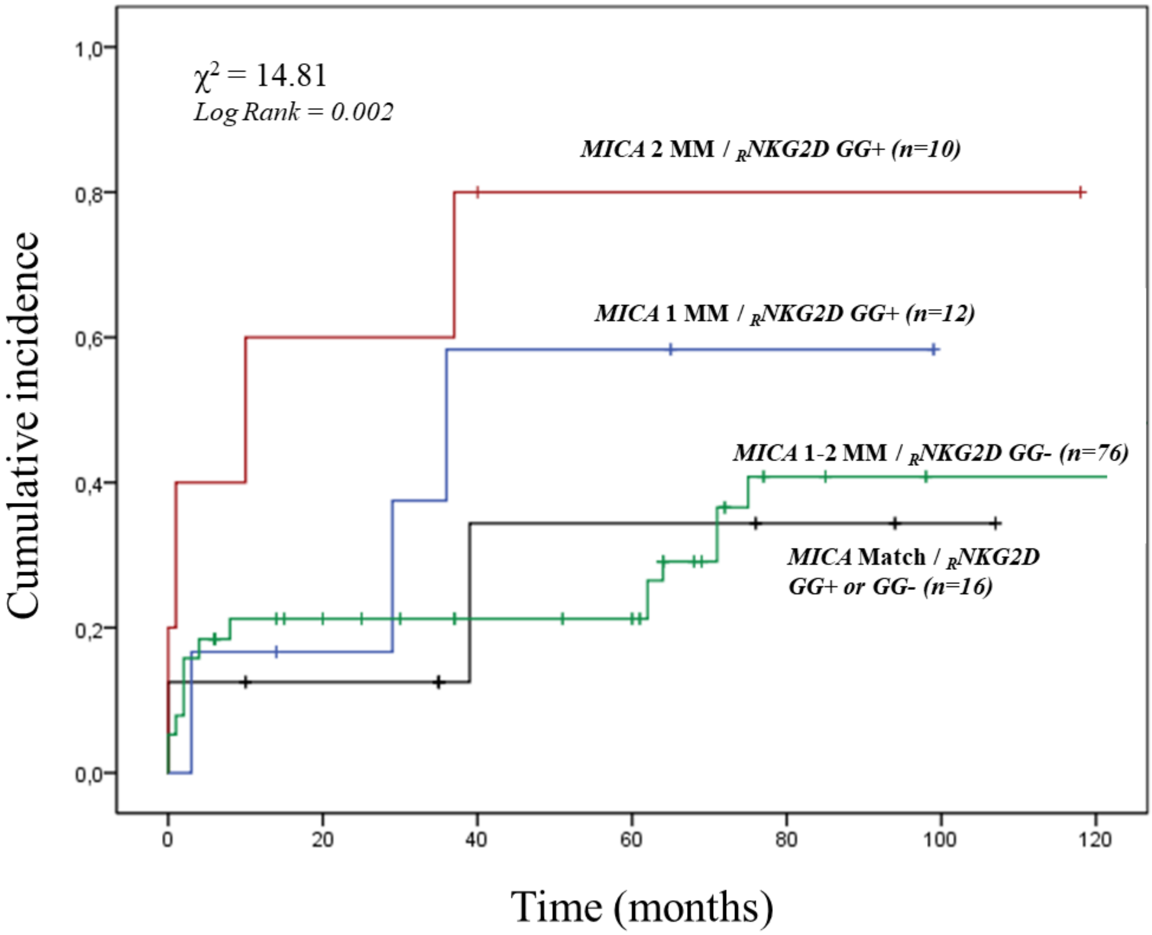

Time (Months)	0	20	40	60	80	100	120	
MICA 2 MM / *NKG2D GG+*	9	4	2	1	1	1	0	Number at risk
MICA 1 MM / *NKG2D GG+*	12	84	4	2	2	0	0	
MICA 1-2 MM / *NKG2D GG-*	76	52	36	34	12	8	8	
MICA Match / *NKG2D GG+ or GG-*	16	12	6	6	4	2	0	

Figure S11

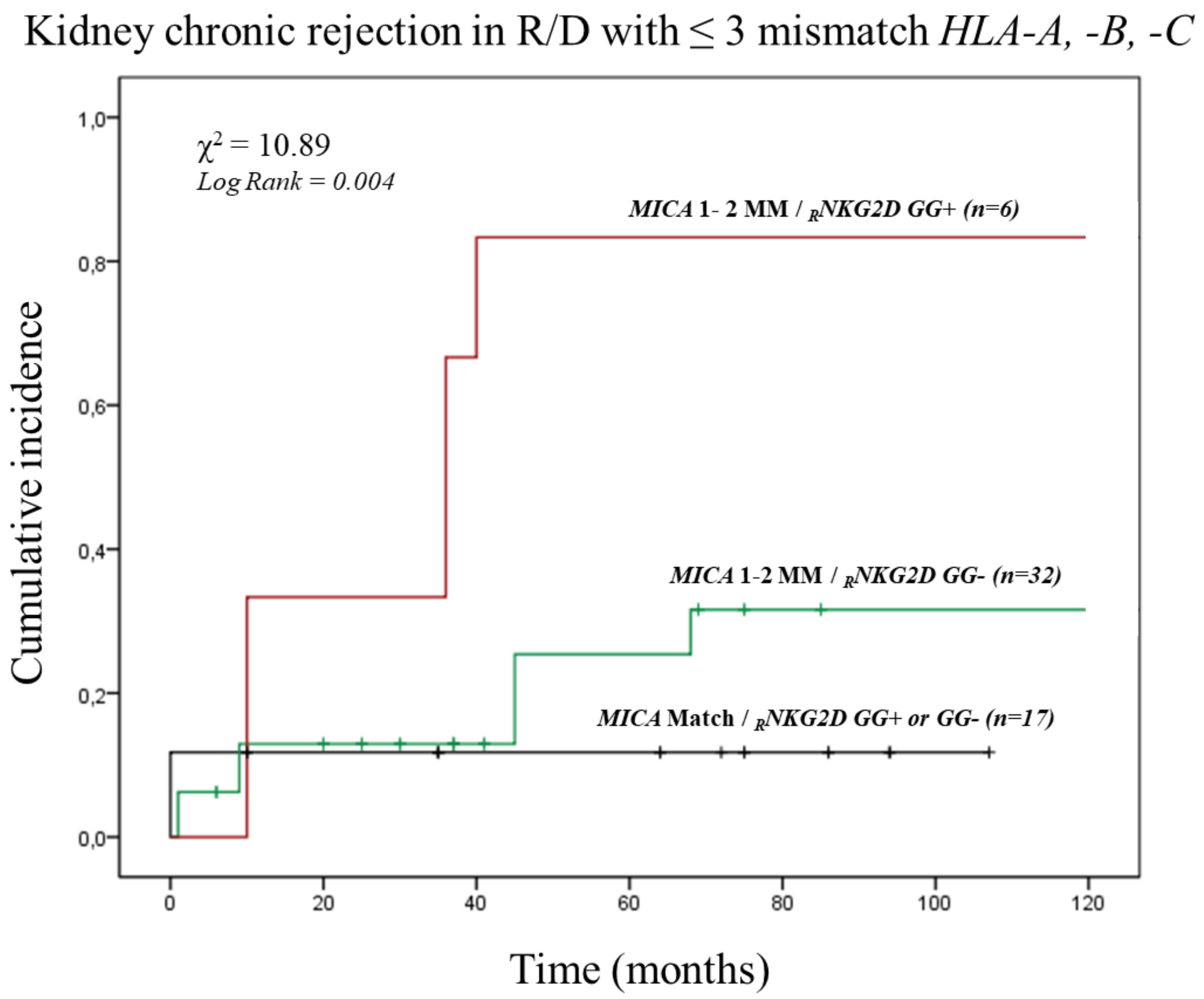

Time (Months)	0	20	40	60	80	100	120	
MICA 1- 2 MM / NKG2D GG+	6	4	2	1	1	1	1	**Number at risk**
MICA 1-2 MM / NKG2D GG-	32	26	16	12	7	5	5	
MICA Match / NKG2D GG+ or GG-	17	13	10	10	5	1	0	

Figure S12

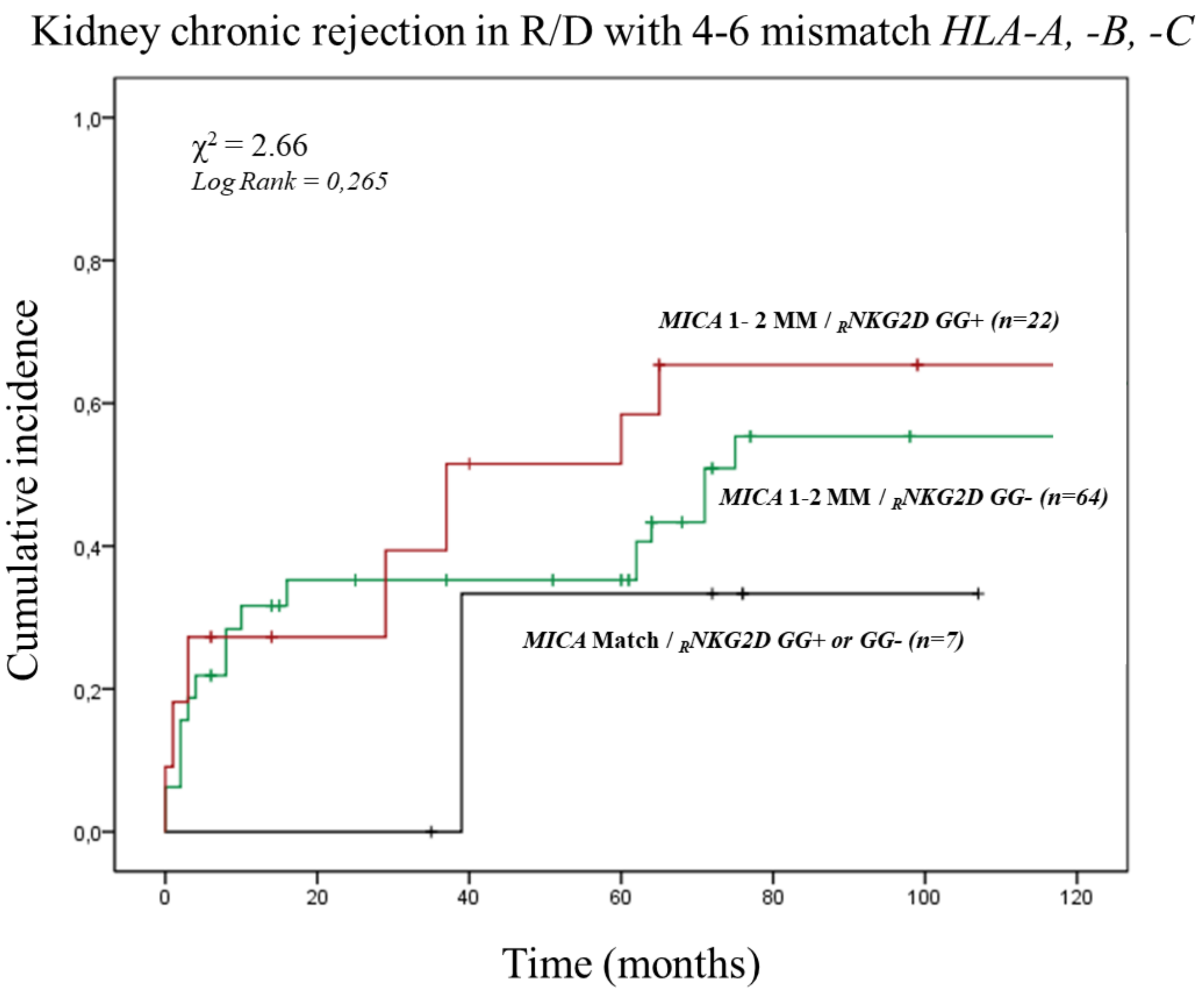

Time (Months)	0	20	40	60	80	100	120	
MICA 1- 2 MM / NKG2D GG+	22	12	8	7	3	1	0	**Number at risk**
MICA 1-2 MM / NKG2D GG-	64	36	30	28	8	6	6	
MICA Match / NKG2D GG+ or GG-	7	7	4	4	1	1	0	

Supplementary Table

Table S1: **RefSNP ID position and primer data.**

RefSNP ID	Position (GRCh38)	Gene	Primer name	Sequence	Length	Tm	GC%
rs1049174	**12:10372766**	***KLRK1 (NKG2D)***	rs1049174 _For	TTCAGATATCCCCAAGGCTGC	21	59.86	52.38
			rs1049174 _Rev	TTAAGATAATCATGGCCCACTGG	23	58.27	43.48
rs2255336	**12:10379727**	***KLRK1 (NKG2D)***	rs2255336 _For	TTCTGCTGCTTCATCGCTGTA	21	60.07	47.62
			rs2255336 _Rev	CAAGGGAATTTGAACTTCTTGGT	23	57.46	39.13

Tm = Primer melting temperature
GC% = Guanine and Cytosine content percentage

Tables S2 Linkage Disequilibrium. Number of observed and expected genotypes consisting of *rs1049174* G>C alleles or/and *rs2255336* A>G alleles. The parameter D, difference between the observed and expected frequencies, is a measure of the linkage disequilibrium. D' is D normalized to one. The parameter r^2 is the squared correlation between the alleles at two loci. The χ^2 values express the discrepancy between the observed and expected frequencies in each cohort. The P values for the comparison between genotypes in the patient and control groups or in the SGF and ABMR cohorts were obtained from the chi-square variable corresponding to the difference between the χ^2 values in the two groups. The P values for the comparison between alleles were computed using Fisher's two-tailed exact test.

A

		**Healthy controls (2N = 292)**							**Kidney transplant patients (2N = 296)**							**Controls vs patients**
		**Observed**	**Expected**						**Observed**	**Expected**						
**SNP**	**Genotypes**	***n (%)***	***n (%)***	***D* (%)**	***D'***	r^2	χ^2	***P***	***n (%)***	***n (%)***	***D* (%)**	***D'***	r^2	χ^2	***P***	**P value**
rs1049174 G>C	GC	67 (22.95)	65 (22.41)	0.54	0.05	0.0006	0.01	0.921	54 (18.24)	72 (24.23)	-5.99	-0.25	0.0610	2.91	0.088	0.234
	GG	16 (5.48)	17 (5.75)	-0.27	-0.05	0.0002	0	1	34 (11.49)	25 (8.49)	3.00	0.25	0.0226	1.20	0.272	0.547
	CC	63 (21.58)	64 (21.84)	-0.26	-0.01	0.0001	0	1	60 (20.27)	51 (17.28)	2.99	0.25	0.0178	0.71	0.400	0.701
rs2255336 A>G	AG	67 (22.95)	60 (20.64)	2.31	0.27	0.0125	0.36	0.547	68 (22.97)	63 (21.42)	1.55	0.16	0..53	0.16	0.692	1
	AA	9 (3.08)	12 (4.24)	-1.16	-0.27	0.0052	0.20	0.657	12 (4.05)	14 (4.83)	-0.78	-0.16	0.0021	0.04	0.841	1
	GG	70 (23.97)	73 (25.13)	-1.16	-0.06	0.0028	0.04	0.847	68 (22.97)	70 (23.75)	-0.78	-0.04	0.0013	0.01	0.923	1
rs1049174 G>C and *rs2255336*	CA								48 (16.22)	54 (18.27)	-2.05	-0.11	0.0081	0.30	0.586	
	CG								114 (38.51)	120 (40.51)	-2.00	-0.16	0.0077	0.18	0.674	

A>G	GA								76 (25.68)	38 (12.81)	12.87	0.70	0.3189	14.87	**0.0001**	
	GG								76 (25.68)	84 (28.41)	-2.73	-0.15	0.0144	0.42	0.517	
	**Alleles**															
rs1049174 G>C	G	99 (33.90)							122 (41.22)							0.074
	C	193 (66.10)							174 (58.78)							0.074
rs2255336 A>G	A	85 (29.11)							92 (31.08)							0.653
	G	207 (70.89)							204 (68.92)							0.653

B

		SGF (2N = 160)							ABMR (2N = 136)							SGF vs ABMR
		Observed	Expected						Observed	Expected						P value
SNP	Genotypes	*n (%)*	*n (%)*	*D (%)*	*D'*	r^2	χ^2	*P*	*n (%)*	*n (%)*	*D (%)*	*D'*	r^2	χ^2	*P*	
rs1049174 G>C	GC	35 (21.88)	38 (23.59)	-1.71	-0.07	0.0053	0.07	0.790	19 (13.97)	34 (24.74)	-10.77	-0.44	0.1894	4.59	**0.032**	0.104

	GG	13 (8.12)	12 (7.27)	0.85	0.07	0.0020	0	1	21 (15.44)	14 (10.06)	5.38	0.44	0.0673	1.18	0.277	0.554
	CC	32 (20.00)	31 (19.14)	0.86	0.07	0.0015	0	1	28 (20.59)	21 (15.21)	5.38	0.44	0.0586	0.90	0.344	0.639
rs2255336 A>G	AG	42 (26.25)	34 (21.48)	4.77	0.49	0.0492	0.85	0.358	26 (19.12)	29 (21.35)	-2.23	-0.10	0.0109	0.09	0.763	1
	AA	4 (2.50)	8 (4.88)	-2.38	-0.49	0.0201	0.78	0.377	8 (5.88)	6 (4.77)	1.11	0.10	0.0045	0.08	0.784	1
	GG	34 (21.25)	38 (23.63)	-2.38	-0.12	0.0117	0.16	0.688	34 (25.00)	32 (23.89)	1.11	0.10	0.0026	0.02	0.888	1
rs1049174 G>C and *rs2255336 A>G*	CA	33 (20.62)	31 (19.34)	1.28	0.11	0.0033	0.02	0.889	15 (11.03)	23 (17.03)	-6.00	-0.35	0.0682	1.50	0.221	0.477
	CG	67 (41.88)	68 (42.54)	-0.66	-0.06	0.0009	0	1	47 (34.56)	52 (38.12)	-3.56	-0.26	0.0240	0.25	0.614	0.881
	GA	42 (26.25)	19 (11.91)	14.34	0.74	0.4055	9.80	**0.002**	34 (25.00)	19 (13.85)	11.15	0.66	0.2354	4.59	**0.032**	1
	GG	44 (27.50)	42 (26.21)	1.29	0.11	0.0033	0.02	0.900	32 (23.53)	42 (31.00)	-7.47	-0.44	0.1057	1.50	0.220	0.475
	**Alleles**															
rs1049174 G>C	G	61 (38.13)							61 (44.85)							0.286
	C	99 (61.88)							75 (55.15)							0.286

rs2255336 A>G	A	50 (31.25)	42 (30.88)	1
	G	110 (68.75)	94 (69.12)	1

Tables S3: Anti-MICA Alloantibodies (MICA-DSA): Allelic Typing and Antibody Quantification.

MICA Alleles					MICA-DSAs Allontibody (MFI)
**N patient**	**Recipient**		**Donor**		
1	**001:01*	**007:01*	**001:01*	**004:01*	04 (900)
2	**004:01*	**018:01*	**002:01*	**016:01*	02 (<400)
3	**008:04*	**009:01*	**002:01*	**018:01*	02, 18 (900)
4	**002:01*	**011:01*	**008:01*	**009:01*	09 (<400)
5	**004:01*	**016:01*	**001:01*	**008:01*	01, 08 (1400)
6	**016:01*	**018:01*	**008:01*	**018:01*	08 (<400)
7	**001:01*	**008:01*	**007:01*	**007:01*	07 (<400)
8	**001:01*	**012:01*	**002:01*	**012:01*	02 (1000)
9	**004:01*	**008:01*	**002:01*	**002:01*	02 (<400)
10	**001:01*	**009:01*	**002:01*	**007:01*	02 (<400)
11	**002:01*	**004:01*	**002:01*	**018:01*	18 (4300)
12	**008:01*	**008:01*	**001:01*	**018:01*	01, 18 (3500)
13	**002:01*	**002:01*	**002:01*	**009:01*	09 (<400)
14	**001:01*	**001:01*	**002:01*	**018:01*	02, 18 (800)
15	**004:01*	**008:01*	**004:01*	**018:01*	18 (800)
16	**001:01*	**008:01*	**002:01*	**018:01*	18 (1200)
17	**001:01*	**004:01*	**009:02*	**018:01*	18 (900)

List of patients who developed anti-MICA alloantibodies against the recipient (MICA-DSA). The table describes the MICA allelic typing of both the patient and the donor. The quantification of MICA antigen alloantibodies detected was measured using single antigen Luminex bead arrays and expressed as normalized mean fluorescence intensity (MFI).

Abbreviation: MICA-DSA: donor-specific antigen MICA alloantibodies.

Supplementary Table S4: Multivariate analysis of clinical and immunogenetic factors associated with SGF and ABMR in kidney transplanted patients.

**Characteristics of transplanted patients**	**Total patients (N = 148)**	**SGF (N = 80)**	**ABMR (N = 68)**	**Comparisons of ABMR vs SGF**					
				**Univariate Analysis**			**Multivariate analysis**		
				**OR and x_2-x_1***	**95% CI**	**P_U^**	**OR_M and $(x_2$-$x_1)_M$#**	**95% CI_M**	**P_M^**
Donor age, median (IQR)	45.0 (31.0 – 58.0)	37.5 (26.8 – 47.0)	51.0 (42.0 – 62.0)	12.7	7.8 – 17.6	**$1.2 \cdot 10^{-6}$**	13.3	8.0 – 19.0	**$5.1 \cdot 10^{-5}$**
Donor Male gender, n (%)	98 (65.5)	62 (76.3)	36 (52.9)	0.3	0.2 – 0.7	**0.003**	0.4	0.2 – 0.9	**0.026**
Cold ischemia time, mean ± SD	660.2 ± 230.6	590.3 ± 222.9	747.5 ± 211.5	157.2	74.0 – 590.3	**$3.0 \cdot 10^{-4}$**	157.6	157.3 – 158.7	**0.017**
HLA-DRB1 1-2*MM*, n (%)	114 (77.0)	66 (82.5)	48 (70.6)	0.5	0.2 – 1.9	0.116	0.4	0.2 – 1.0	**0.045**
HLA-A, -B, -C > 3*MM*, n (%)	93 (62.8)	45 (56.3)	48 (70.6)	1.9	0.9 – 3.9	0.088	1.7	0.8 – 3.7	0.166
MICA 2*MM*, n (%)	72 (48.6)	36 (45.0)	36 (52.9)	1.4	0.7 – 2.8	0.410	1.6	0.8 – 3.4	0. 189
rs1049174 GG, n (%)	34 (23.0)	13 (16.3)	21 (30.9)	2.3	1.0 – 5.5	**0.049**	1.9	0.8 – 4.7	0.164
rs2255336 AA, n (%)	12 (8.1)	4 (5.0)	8 (11.8)	0.3	0.1 – 0.8	**0.006**	2.4	0.6 – 10.2	0.213
MICA 2*MM* + *rs1049174 GG*, n (%)	12 (8.1)	2 (2.5)	10 (14.7)	6.6	1.3 – 64.7	**0.012**	0.1	0.0 – 0.6	**0.020**

De Novo DSA HLA Class I[†], n (%)	4 (2.7)	0 (0)	4 (5.9)	0.0	0.0 – 1.3	**0.042**	-	-	0.986
De Novo DSA HLA Class II, n (%)	12 (7.4)	2 (0)	10 (16.2)	6.6	1.3 – 64.7	**0.012**	10.6	2.2 – 79.3	**0.007**

^ P_U = P value in univariate analysis; P_M = P value adjusted for age and gender.

* x_2-x_1 = mean difference for continuous variables (donor age and cold ischemia time): x_2 (patients with ABMR) - x_1 (patients with SGF).
